# Long-term gastrointestinal outcomes of COVID-19

**DOI:** 10.1038/s41467-023-36223-7

**Published:** 2023-03-07

**Authors:** Evan Xu, Yan Xie, Ziyad Al-Aly

**Affiliations:** 1 Clinical Epidemiology Center, Research and Development Service, VA Saint Louis Health Care System, Saint Louis, MO USA; 2 Veterans Research and Education Foundation of Saint Louis, Saint Louis, MO USA; 3 Department of Epidemiology and Biostatistics, College for Public Health and Social Justice, Saint Louis University, Saint Louis, MO USA; 4 Department of Medicine, Washington University School of Medicine, Saint Louis, MO USA; 5 Nephrology Section, Medicine Service, VA Saint Louis Health Care System, Saint Louis, MO USA; 6 Institute for Public Health, Washington University in Saint Louis, Saint Louis, MO USA

**Keywords:** Gastrointestinal diseases, Viral infection, Infectious diseases, SARS-CoV-2

## Abstract

A comprehensive evaluation of the risks and 1-year burdens of gastrointestinal disorders in the post-acute phase of COVID-19 is needed but is not yet available. Here we use the US Department of Veterans Affairs national health care databases to build a cohort of 154,068 people with COVID-19, 5,638,795 contemporary controls, and 5,859,621 historical controls to estimate the risks and 1-year burdens of a set of pre-specified incident gastrointestinal outcomes. We show that beyond the first 30 days of infection, people with COVID-19 exhibited increased risks and 1-year burdens of incident gastrointestinal disorders spanning several disease categories including motility disorders, acid related disorders (dyspepsia, gastroesophageal reflux disease, peptic ulcer disease), functional intestinal disorders, acute pancreatitis, hepatic and biliary disease. The risks were evident in people who were not hospitalized during the acute phase of COVID-19 and increased in a graded fashion across the severity spectrum of the acute phase of COVID-19 (non-hospitalized, hospitalized, and admitted to intensive care). The risks were consistent in comparisons including the COVID-19 vs the contemporary control group and COVID-19 vs the historical control group as the referent category. Altogether, our results show that people with SARS-CoV-2 infection are at increased risk of gastrointestinal disorders in the post-acute phase of COVID-19. Post-covid care should involve attention to gastrointestinal health and disease.

## Abstract (web summary)

SARS-CoV-2 infection can lead to varied post-acute symptoms in the lungs and other organs, including the gastrointestinal system. Here the authors estimate the risks and 1-year burdens of a set of pre-specified incident gastrointestinal outcomes following SARS-CoV-2 infection in an electronic health care record-based cohort study.

## Introduction

SARS-CoV-2 infection can lead to a broad array of post-acute sequelae which can involve the pulmonary and several extrapulmonary organs including the gastrointestinal system^1,2^; the constellation of these post-acute conditions is referred to by the umbrella term Long Covid^3^. Studies investigating the gastrointestinal post-acute sequelae of SARS-CoV-2 infection are mostly limited to hospitalized individuals and all had a short duration of follow up of a few months, and a narrow selection of gastrointestinal outcomes^4–12^. A comprehensive evaluation of the risks and burdens of gastrointestinal disorders in the post-acute phase of COVID-19 is needed but has not yet been undertaken. Addressing this knowledge gap is important to inform post-acute COVID-19 care strategies.

In this work, we use the US Department of Veterans Affairs national health care databases to build a cohort of 154,068 people who survived the first 30 days of COVID-19, and two control groups including a contemporary control of 5,638,795 who lived during the same time but had no evidence of SARS-CoV-2 infection, and a historical cohort of 5,859,621 people from the pre-pandemic era. These cohorts were followed longitudinally to estimate the risks and 1-year burdens of a set of pre-specified incident gastrointestinal outcomes in the overall cohort and by care setting of the acute phase of SARS-CoV-2 infection (that is whether people were non-hospitalized, hospitalized, or admitted to intensive care).

## Results

The COVID-19, contemporary control, and historical control group consisted of 154,068, 5,638,795, and 5,859,621 participants, respectively. The COVID-19, contemporary control, and historical control groups had a median follow up of 408 (interquartile range: 378–500), 409 (379–505), and 409 (379–504) days, which corresponded to 185,399, 6,808,464, and 7,071,123 person-years of follow-up, respectively; altogether totaling to 14,064,985 person-years of follow-up.

Baseline demographic and health characteristics of the COVID-19, contemporary control, and historical control groups are presented in supplementary table 1. Characteristics after weighting are presented in supplementary table 2.

### Incident gastrointestinal outcomes in COVID-19 vs contemporary controls

The COVID-19 and contemporary control groups were balanced through the inverse probability weighting method; examination of standardized mean differences of demographic and health characteristics after weighting suggested good balance (supplementary fig. 1a).

**Fig. 1:**
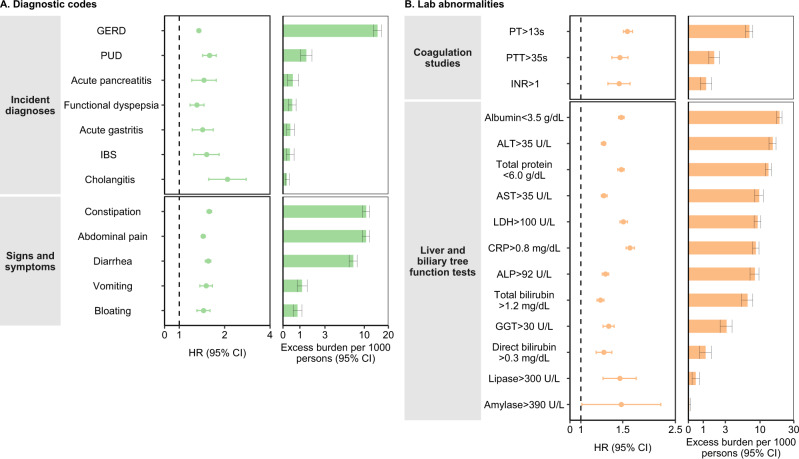
Risks and 1-year burdens of incident post-acute COVID-19 gastrointestinal outcomes compared with the contemporary control cohort. Outcomes were ascertained 30 d after the COVID-19-positive test until the end of follow-up. COVID-19 cohort (*n*  =  154,068) and contemporary control cohort (*n*  =  5,638,795). Panel **A** describes the risks and burdens of incident diagnoses (light green) and panel **B** describes the risks and burdens of incident laboratory abnormalities (orange). Adjusted HRs (dots) and 95% (error bars) CIs are presented, as are estimated excess burdens (bars) and 95% CIs (error bars). Burdens are presented per 1000 persons at 12 months of follow up. The dashed line marks a HR of 1.00; lower limits of 95% CIs with values greater than 1.00 indicate significantly increased risk. GERD, gastroesophageal reflux disorder; IBS irritable bowel syndrome, PT prothrombin time, PTT partial thromboplastin time, INR international normalized ratio, ALT alanine transaminase, AST aspartate transaminase, LDH lactate dehydrogenase, CRP c-reactive peptide, ALP alkaline phosphatase, GGT γ-glutamyl transferase.

**Fig. 2:**
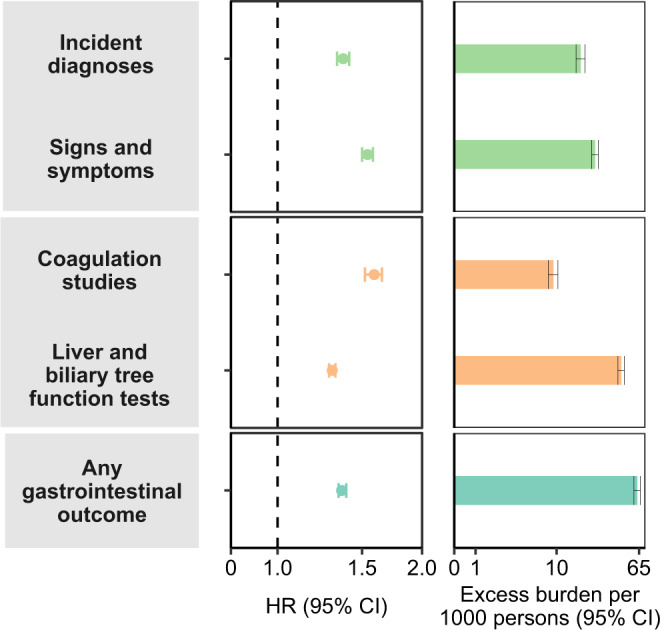
Risks and 1-year burdens of incident post-acute COVID-19 composite gastrointestinal outcomes compared with the contemporary control cohort. Composite outcomes consisted of incident diagnoses (GERD, PUD, acute pancreatitis, functional dyspepsia, acute gastritis, IBS, and cholangitis), signs and symptoms (constipation, abdominal pain, diarrhea, vomiting, and bloating), coagulation studies (PT, PTT, INR), liver and biliary tree function tests (albumin, ALT, total protein, AST, LDH, CRP, ALP, total bilirubin, GGT, direct bilirubin, lipase, and amylase) and any gastrointestinal outcome (incident occurrence of any gastrointestinal outcome studied). Outcomes were ascertained 30d after the COVID-19-positive test until the end of follow-up. COVID-19 cohort (*n*  =  154,068) and contemporary control cohort (*n*  =  5,638,795). Adjusted HRs (dots) and 95% (error bars) CIs are presented, as are estimated excess burdens (bars) and 95% CIs (error bars). Burdens are presented per 1000 persons at 12 months of follow up. The dashed line marks a HR of 1.00; lower limits of 95% CIs with values greater than 1.00 indicate significantly increased risk. GERD gastroesophageal reflux disorder, IBS irritable bowel syndrome, PT prothrombin time, PTT partial thromboplastin time, INR international normalized ratio, ALT alanine transaminase, AST aspartate transaminase, LDH lactate dehydrogenase, CRP c-reactive peptide, ALP alkaline phosphatase, GGT γ-glutamyl transferase.

We estimated the risks (hazard ratios; HR) and excess burdens of a set of prespecified gastrointestinal outcomes in people with COVID-19 vs the contemporary control group (reference category) (Figs. 1, 2 and supplementary table 3). The excess burdens were estimated per 1000 persons at 1-year based on the difference in the estimated incidence rate between the COVID-19 and contemporary control group at 1 year.

#### Incident diagnoses

People who survived the first 30 days of COVID-19 exhibited increased risk of gastroesophageal reflux disease (GERD) (HR 1.35 (1.31, 1.39); burden 15.50 (13.83, 17.21) per 1000 persons at 1-year); for all hazard ratios and burdens, parenthetical ranges refer to 95% confidence intervals), peptic ulcer disease (PUD) (HR 1.62 (1.46, 1.79); burden 1.57 (1.18, 2)), acute pancreatitis (HR 1.46 (1.23, 1.75); burden 0.6 (0.29, 0.97)), functional dyspepsia (HR 1.36 (1.22, 1.51); burden 0.63 (0.39, 0.9)), acute gastritis (HR 1.47 (1.25, 1.72); burden 0.47 (0.26, 0.73)), irritable bowel syndrome (HR 1.54 (1.28, 1.86); burden 0.44 (0.22, 0.69)), and cholangitis (HR 2.02 (1.55, 2.63); burden 0.22 (0.12, 0.35)). The risk and burden of a composite of any incident diagnosis were 1.37 (1.33, 1.41) and 17.37 (15.62, 19.17).

#### Signs and symptoms

included constipation (HR 1.60 (1.54, 1.66); burden 11.34 (10.27, 12.46)), abdominal pain (HR 1.44 (1.4, 1.49); burden 10.53 (9.42, 11.67)), diarrhea (HR 1.58 (1.52, 1.65); burden 7.99 (7.13, 8.9)), vomiting (HR 1.52 (1.38, 1.67)); burden 1.21 (0.90, 1.56)), and bloating (HR 1.46 (1.33, 1.61); burden 0.92 (0.65, 1.21)). The risk and burden of a composite of these signs and symptoms were 1.54 (1.5, 1.58), and 24.02 (22.30, 25.78).

#### Coagulation studies

included prothrombin time>13 s (HR 1.61 (1.54, 1.68); burden 7.90 (6.99, 8.84)); partial thromboplastin time>35 s (HR 1.49 (1.38, 1.61); burden 2.11 (1.63, 2.64)), and international normalized ratio>1 (HR 1.48 (1.33, 1.65); burden 1.37 (0.93, 1.85)). The risk and burden of a composite of these coagulation studies were 1.59 (1.52, 1.65), and 9.27 (8.27, 10.31).

#### Liver and biliary tree function tests

included albumin<3.5 g/dL (HR 1.50 (1.46, 1.54); burden 20.9 (19.27, 22.57)); alanine transaminase>35 U/L (HR 1.25 (1.23, 1.28); burden 16.39 (14.60, 18.22)); total protein<6.0 g/dL (HR 1.50 (1.45, 1.54)); burden 14.54 (13.26, 15.85)), aspartate transaminase>35 U/L (HR 1.27 (1.23, 1.30); burden 10.69 (9.33, 12.10)), lactate dehydrogenase>100 U/L (HR 1.54 (1.48, 1.60); burden 10.22 (9.17, 11.31)), c-reactive peptide>0.8 mg/dL (HR 1.63 (1.56, 1.69); 9.43 (8.49, 10.40)), alkaline phosphatase>92 U/L (HR 1.28 (1.25, 1.33); burden 9.21 (7.94, 10.52)), total bilirubin>1.2 mg/dL (HR 1.22 (1.18, 1.26); burden 7.40 (6.11, 8.73)), γ-glutamyl transferase>30 U/L (HR 1.30 (1.24, 1.37); burden 3.25 (2.54, 3.99)), direct bilirubin>0.3 mg/dL (HR 1.30 (1.20, 1.40); burden 1.43 (0.98, 1.92)), lipase>95 U/L (HR 1.49 (1.27, 1.74); burden 0.54 (0.30, 0.82)) and amylase>130 U/L (HR 1.54 (1.05, 2.26); burden 0.07 (0.01, 0.16)). The risk and burden of a composite of these liver and biliary tree function tests were 1.30 (1.28, 1.32), and 43.49 (40.30, 46.72).

#### Any gastrointestinal outcome

Compared to the contemporary control group, the risk of having any gastrointestinal outcome (defined as the occurrence of any incident prespecified gastrointestinal outcome included in this study) was increased in COVID-19 group (HR 1.36 (1.34, 1.39); burden 62.34 (57.82, 66.92)).

#### Subgroup analyses

**Fig. 3:**
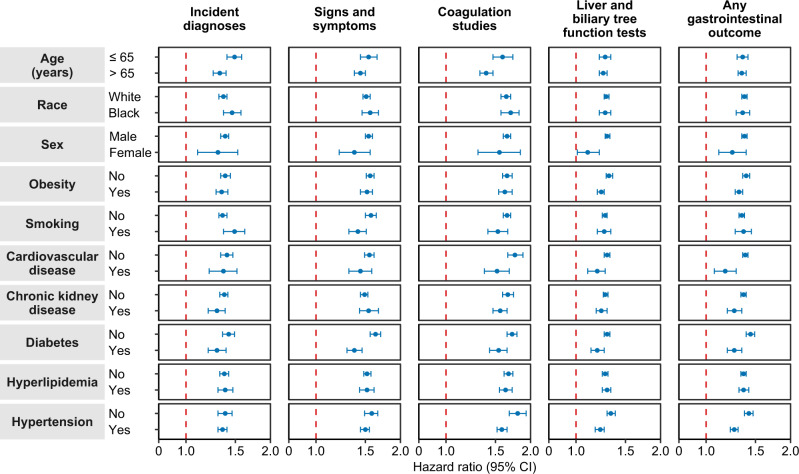
Subgroup analyses of the risks of incident post-acute COVID-19 composite gastrointestinal outcomes compared with the contemporary control cohort. Composite outcomes consisted of incident diagnoses (GERD, PUD, acute pancreatitis, functional dyspepsia, acute gastritis, IBS, and cholangitis), signs and symptoms (constipation, abdominal pain, diarrhea, vomiting, and bloating), coagulation studies (PT, PTT, INR), liver and biliary tree function tests (albumin, ALT, total protein, AST, LDH, CRP, ALP, total bilirubin, GGT, direct bilirubin, lipase, and amylase) and any gastrointestinal outcome (incident occurrence of any gastrointestinal outcome studied). Outcomes were ascertained 30d after the COVID-19-positive test until the end of follow-up. COVID-19 cohort (*n*  =  154,068) and contemporary control cohort (*n*  =  5,638,795). Adjusted HRs (dots) and 95% (error bars) CIs are presented. The dashed line marks a HR of 1.00; lower limits of 95% CIs with values greater than 1.00 indicate significantly increased risk. GERD gastroesophageal reflux disorder, IBS irritable bowel syndrome, PT prothrombin time, PTT partial thromboplastin time, INR international normalized ratio, ALT alanine transaminase, AST aspartate transaminase, LDH lactate dehydrogenase, CRP c-reactive peptide, ALP alkaline phosphatase, GGT γ-glutamyl transferase.

We examined the risks of composite gastrointestinal outcomes in COVID-19 group compared to contemporary control in prespecified subgroups. The results showed that the risks of composite gastrointestinal outcome were evident in all subgroups based on age, race, sex, obesity, smoking, cardiovascular disease, chronic kidney disease, diabetes, hyperlipidemia, and hypertension (Fig. 3 and supplementary table 4).

### Incident gastrointestinal outcomes in COVID-19 vs contemporary controls by care setting of the acute infection

We then stratified the COVID-19 cohort into mutually exclusive groups based on the care setting of the acute infection comprised of those who were non-hospitalized (*n* = 131,915), hospitalized (*n* = 16,764), or admitted to intensive care (*n* = 5389) during the acute phase of COVID-19. The risks and burdens of prespecified gastrointestinal outcomes were then estimated. Demographic and health characteristic data of these three groups before and after weighting are described in supplementary tables 5 and 6, respectively. After application of inverse probability weighting, calculation of standardized mean differences of demographic and health characteristics suggested good balance (Supplementary fig. 1b).

**Fig. 4:**
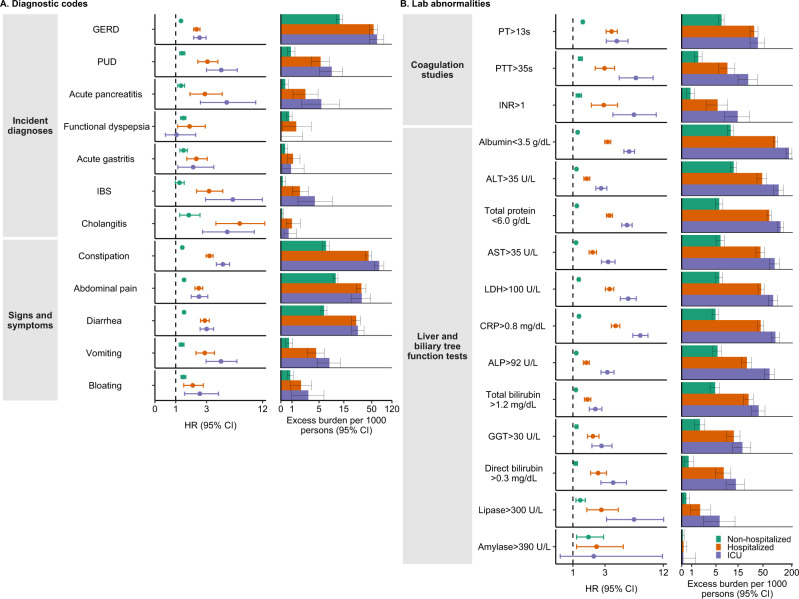
Risks and 1-years burdens of incident post-acute COVID-19 gastrointestinal outcomes compared with the contemporary control cohort by care setting of the acute infection. Risks and burdens were assessed at 1-year in mutually exclusive groups comprising non-hospitalized individuals with COVID-19 (green), individuals hospitalized for COVID-19 (orange) and individuals admitted to intensive care for COVID-19 during the acute phase (first 30 d) of COVID-19 (blue). Outcomes were ascertained 30 d after the COVID-19-positive test until the end of follow-up. The contemporary control cohort served as the referent category. Within the COVID-19 cohort, non-hospitalized (*n*  =  131,915), hospitalized (*n*  =  16,764), admitted to intensive care (*n*  =  5389) and contemporary control cohort (*n*  =  5,606,761). Panel **A** describes the risks and burdens of incident diagnoses and panel **B** describes the risks and burdens of incident laboratory abnormalities. Adjusted HRs (dots) and 95% (error bars) CIs are presented, as are estimated excess burdens (bars) and 95% CIs (error bars). Burdens are presented per 1000 persons at 12 months of follow up. The dashed line marks a HR of 1.00; lower limits of 95% CIs with values greater than 1.00 indicate significantly increased risk. GERD gastroesophageal reflux disorder, IBS irritable bowel syndrome, PT prothrombin time, PTT partial thromboplastin time, INR international normalized ratio, ALT alanine transaminase, AST aspartate transaminase, LDH lactate dehydrogenase, CRP c-reactive peptide, ALP alkaline phosphatase, GGT γ-glutamyl transferase.

**Fig. 5:**
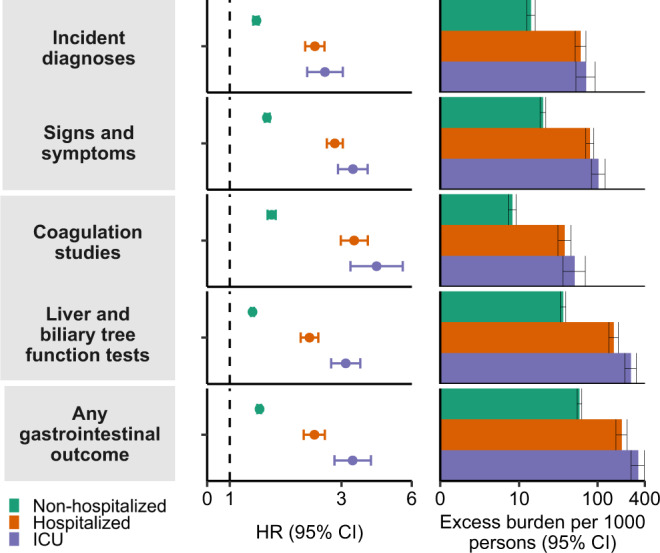
Risks and 1-year burdens of incident post-acute COVID-19 composite gastrointestinal outcomes compared with the contemporary control cohort by care setting of the acute infection. Risks and burdens were assessed at 1-year in mutually exclusive groups comprising non-hospitalized individuals with COVID-19 (green), individuals hospitalized for COVID-19 (orange) and individuals admitted to intensive care for COVID-19 during the acute phase (first 30 d) of COVID-19 (blue). Composite outcomes consisted of incident diagnoses (GERD, PUD, acute pancreatitis, functional dyspepsia, acute gastritis, IBS, and cholangitis), signs and symptoms (constipation, abdominal pain, diarrhea, vomiting, and bloating), coagulation studies (PT, PTT, INR), liver and biliary tree function tests (albumin, ALT, total protein, AST, LDH, CRP, ALP, total bilirubin, GGT, direct bilirubin, lipase, and amylase) and any gastrointestinal outcome (incident occurrence of any gastrointestinal outcome studied). Outcomes were ascertained 30 d after the COVID-19-positive test until the end of follow-up. The contemporary control cohort served as the referent category. Within the COVID-19 cohort, non-hospitalized (*n*  =  131,915), hospitalized (*n*  =  16,764), admitted to intensive care (*n*  =  5389) and contemporary control cohort (*n*  =  5,606,761). Adjusted HRs (dots) and 95% (error bars) CIs are presented, as are estimated excess burdens (bars) and 95% CIs (error bars). Burdens are presented per 1000 persons at 12 months of follow up. The dashed line marks a HR of 1.00; lower limits of 95% CIs with values greater than 1.00 indicate significantly increased risk. GERD gastroesophageal reflux disorder, IBS, irritable bowel syndrome, PT prothrombin time, PTT partial thromboplastin time, INR international normalized ratio, ALT alanine transaminase, AST aspartate transaminase, LDH lactate dehydrogenase, CRP c-reactive peptide, ALP alkaline phosphatase, GGT γ-glutamyl transferase.

Compared to the contemporary control group, the risks and burdens of the prespecified gastrointestinal outcomes were evident even among those who were not hospitalized during the acute phase of COVID-19 and increased in a graded fashion according to the care setting of the acute phase of the disease from non-hospitalized to hospitalized to those admitted to intensive care (Fig. 4, Fig. 5, and Supplementary table 7).

### Incident gastrointestinal outcomes in COVID-19 vs historical controls

We tested the robustness of our study results by evaluating the associations between COVID-19 and the prespecified gastrointestinal outcomes in analyses utilizing a historical control group (which did not experience the pandemic) as the referent group. Demographic and health characteristics before and after weighting are presented in supplementary tables 1, 2, 8, and 9. Calculation of the standard mean differences after application of inverse weighting suggested that covariates were well balanced (Supplementary fig. 1c, d). Our results indicated increased risks and burdens of the prespecified outcomes in analyses comparing the overall COVID-19 and the historical control groups (Supplementary figs. 2, 3, and supplementary table 10), in analyses of subgroups (Supplementary fig. 4 and supplementary table 11), and analyses by care setting of the acute phase of COVID-19 (Supplementary figs. 5, 6 and supplementary table 12). Both the direction and the magnitude of risks and burdens were consistent with results from analyses using the contemporary control as the referent category.

### Incident gastrointestinal outcomes in hospitalized COVID-19 vs hospitalized influenza

Additionally, we evaluated the risk of incident composite gastrointestinal outcomes in people hospitalized with COVID-19 (*n* = 22,153) and those hospitalized for a seasonal influenza infection (*n* = 11,050). Compared to seasonal influenza, COVID-19 was associated with an increased risk of abnormal coagulation studies, abnormal liver function tests, and the composite outcome any gastrointestinal outcome (supplementary table 13). While the hazard ratios for the other outcomes (coagulation studies and liver function tests) were above one, the 95% confidence intervals crossed 1 suggesting insufficient precision to reject the null hypothesis.

### Sensitivity analyses

To further test the robustness of our results, we conducted several sensitivity analyses testing the outcome of having any gastrointestinal disorder in comparisons involving the COVID-19 groups and the contemporary control and – separately – the COVID-19 group and the historical control, and additionally in analyses by care setting of the acute phase of COVID-19 and both control groups. 1) We tested the results of models specified to include only predefined covariates (that is, no algorithmically selected high dimensional covariates were used to build the inverse probability weight); 2) we expanded the algorithmic high dimensional covariate selection to include 300 high dimensional covariates to build the inverse probability weight (whereas the primary approach included 100 algorithmically selected high dimensional variables); 3) we employed the doubly robust method through application of both weighting and covariate adjustment in the survival models (instead of the inverse weighting method used in the primary analyses) as an alternative approach to examine the associations between COVID-19 and the risk of the prespecified gastrointestinal outcomes. The results from all sensitivity analyses were consistent with those generated using the primary approach and are presented in supplementary tables 14 and 15.

### Positive and negative outcome controls

To assess whether our approach would reproduce known association, we tested fatigue as a positive outcome control. Our results showed that COVID-19 was associated with increased risk of fatigue in comparisons vs the contemporary and historical control groups (supplementary table 16).

We then subjected our analytic approach to testing a set of 4 negative outcome controls where prior knowledge suggests no expected associations. Consistent with a priori expectations, the results showed no significant association between COVID-19 and any of the negative outcome controls in comparisons with both the contemporary and historical control groups (supplementary table 16).

### Negative exposure control

To further test the rigor of our approach, we examined the associations between a pair of negative exposure controls and our prespecified gastrointestinal outcomes. We hypothesized that receiving the influenza vaccination on odd- vs even- numbered calendar days between March 1, 2020 and January 15, 2021 would not be associated with either increased or decreased risk of each of the prespecified outcomes utilized in this analysis. We therefore tested the associations between receiving the influenza vaccine in even- (*n* = 571,291) vs odd- (*n* = 605,453) numbered calendar days and the prespecified gastrointestinal outcomes. Data sources, cohort design, analytic approach (encompassing covariate specification and weighting method), and prespecified outcomes were identical to those used in the primary analysis. Consistent with pre-test expectations, our results indicated that receiving the influenza vaccination in odd-numbered vs even-numbered calendar days was not significantly associated with any of the prespecified outcomes (Supplementary table 17).

## Discussion

In this work involving 11,652,484 people including 154,068 people with COVID-19, 5,638,795 contemporary controls, and 5,859,621 historical controls — which altogether correspond to 14,064,985 person years of follow up, we provide evidence that beyond the first 30 days of infection, people with COVID-19 exhibited increased risks and 1-year burdens of incident gastrointestinal disorders spanning several disease categories including motility disorders, acid related disorders (dyspepsia, GERD, PUD), functional intestinal disorders, acute pancreatitis, hepatic and biliary disease. The risks were evident in subgroups based on age, race, sex, obesity, smoking, cardiovascular disease, chronic kidney disease, diabetes, hyperlipidemia and hypertension. The risks were evident in people who were not hospitalized during the acute phase of COVID-19 and increased in a graded fashion across the severity spectrum of the acute phase of COVID-19 (from non-hospitalized to hospitalized individuals, to those admitted to intensive care). The risks were consistent in comparisons including the COVID-19 vs the contemporary control group and COVID-19 vs the historical control group as the referent category. A comparative analysis suggested that those hospitalized with COVID-19 are at increased risk of several gastrointestinal outcomes compared to those hospitalized with seasonal influenza. The results were consistently robust to challenge in several sensitivity analyses; and examination of a positive outcome control, a battery of negative outcome controls, and a pair of exposure controls yielded results consistent with pre-test expectations. The constellation of findings suggests that people with SARS-CoV-2 infection are at increased risk of gastrointestinal disorders in the post-acute phase of COVID-19. The risks and burdens are not trivial – suggesting that post-acute covid care strategies should include attention to gastrointestinal disease.

Our findings suggest that gastrointestinal disease is another facet of the multifaceted Long Covid^2,13,14^. The risks were evident even in people whose acute disease did not necessitate hospitalization. This group represents the majority of people with COVID-19. Although the absolute burdens (expressed per 1000 persons at 1-year) may appear small, because of the large number of people with SARS-CoV-2 infection, these rates may translate into large number of affected people. This will have ramifications not only for the personal health of affected individuals, but also on health systems which will have to address the care needs of people with post-acute COVID-19 gastrointestinal disorders^13,14^.

Beyond the acute phase, SARS-CoV-2 infection is associated with increased risk of post-acute sequelae in several organ systems including — as we report here — the gastrointestinal system^1,15–21^. Evidence from other work suggests that vaccines reduce but do not completely abrogate the risk of post-acute sequelae and that reinfection (even in vaccinated individuals) contributes additional risks of health sequelae in both the acute and post-acute phase^22,23^. Altogether the evidence base reinforces the need for continued emphasis on primary prevention of SARS-CoV-2 infection (and prevention of reinfection) as the foundation of the public health response. Woven together with the evidence amassed thus far on the scale and breadth of organ dysfunction in Long Covid, the findings in this report call for the urgent need to develop strategies to prevent and treat the post-acute sequelae of SARS-CoV-2 infection^13^.

Our comparative analyses showing increased risk of gastrointestinal outcomes in people hospitalized with COVID-19 and those hospitalized with seasonal influenza —are useful to benchmark the risk against a well characterized respiratory viral infection^24^.

Several hypotheses have been proposed to explain the myriad manifestations of Long Covid including gastrointestinal manifesations^25–27^. These mechanisms include intestinal microbiome dysbiosis, persistence of the virus in immune privileged sites and subsequent chronic inflammation that may provoke organ injury, autoimmune mechanisms, and tissue injury during the acute phase which lead to clinical sequelae in the post-acute phase of the disease^25,27–37^. Other putative mechanisms may involve the angiotensin-converting enzyme 2 which is constitutively expressed on the brush border of the small intestinal mucosa and several other gastrointestinal cell types^27^. An emerging body of evidence suggests SARS-CoV-2 liver and other gastrointestinal tissue SARS-CoV-2 tropism, residual viral antigens in gastrointestinal and hepatic tissues, persistence of the virus in gastrointestinal tract reservoirs, and ongoing viral replication in the appendix in the post-acute phase of the disease and alteration of gut microbiota in people with Long Covid^30,34,38–43^. Studies integrating multi-dimensional immune phenotyping and machine learning suggested that compared to matched controls, people with Long Covid had increased levels of humoral responses directed against SARS-CoV-2, elevated antibody responses directed against Epstein-Barr virus and elevated cortisol levels but not autoantibodies to human exoproteome – altogether suggesting that persistent antigen, reactivation of latent herpesviruses, and chronic inflammation may be key mechanisms for Long Covid^33^. Despite remarkable progress, a better and deeper understanding of the biologic mechanisms of the post-acute sequelae of SARS-CoV-2 is needed to identify potential intervention opportunities to prevent and treat the condition.

We conceptualize Long Covid using a counterfactual approach — comparing those with SARS-CoV-2 infection versus those without — which allows us to assess all the consequences of SARS-CoV-2 infection. As such the post-acute gastrointestinal sequelae catalogued in this report may represent de novo disease, acceleration of underlying preclinical disease, or adverse treatment effects of SARS-CoV-2. However, regardless of the mechanistic pathway, these post-acute sequelae still represent consequences of the infection and may not have materialized at all or may not have materialized so soon without an infection with SARS-CoV-2.

This study has several strengths. We leveraged the breadth and depth of the national healthcare databases of the US Department of Veterans Affairs to build a large cohort of people with COVID-19. We evaluated the risk of a set of pre-specified outcomes versus two control groups - a contemporary control and a historical control group. We adjusted through inverse weighting for a large set of predefined covariates (specified based on prior knowledge) which were complemented by a set of algorithmically selected covariates from several data domains including diagnostic codes, medications, and laboratory test results. We examined risks and burdens across care settings of the acute infection (non-hospitalized, hospitalized and admitted to intensive care). The results were robust to challenge in several sensitivity analyses, and our approach withstood the scrutinous application of positive and negative outcome controls and negative exposure controls. Finally, we estimated risks based on the relative scale (hazard ratio), and also on the absolute scale (burden) which (because it also incorporates baseline risk) provides more meaningful assessment of population-level risk than risk on the ratio scale (e.g., hazard ratio).

This study has several limitations. The demographic characteristics of our cohorts (majority male) may limit generalizability of the results; however, because of the large size, and although the proportion is tilted toward males, the number of female cohort participants were 1,153,894. We leveraged the vast databases of the US Department of Veterans Affairs to conduct this study, and although we used validated outcome definitions, and took care to adjust the analyses for a large set of predefined and algorithmically selected variables, we cannot completely rule out misclassification bias and residual confounding that may bias study results. It is possible that some cohort participants may have had SARS-CoV-2 infection but were not tested for it and as a result these participants would have been included in the contemporary control group and may have biased the results in favor of the null hypothesis; however, our findings were also consistent in comparisons vs the historical control group from an era that predates the pandemic (when SARS-CoV-2 infection in humans had not been reported). As the pandemic continues, it is likely that further mutations of the virus, increased uptake of vaccine and antivirals, and waning vaccine immunity may result in changes in the epidemiology of the post-acute gastrointestinal sequelae of SARS-CoV-2 infection^44^.

In sum, in this study of 154,068 people who survived the acute phase of COVID-19, we show increased risk and burden of post-acute gastrointestinal sequelae spanning several disease categories including acid disorders, functional intestinal disorders, pancreatic disorders, hepatic and biliary disease. The risks were evident even among those whose acute COVID-19 did not necessitate hospitalization. Our findings suggest that post-acute COVID-19 care strategies should include attention to gastrointestinal health and disease.

## Methods

### Ethics statement

This study was approved by the institutional review board (IRB) of the VA St. Louis Health Care System; because of the observational and retrospective nature of the study, the IRB granted a waiver of informed consent (protocol number 1606333). Participants were not compensated.

### Setting

Data from the US Department of Veterans Affairs’ electronic healthcare databases was utilized in this study. The Veterans Health Administration (VHA) is a branch of the US Department of Veterans affairs; VHA operates the largest nationally integrated healthcare system in the US which is comprised of 1255 healthcare facilities (including 170 VA Medical Centers and 1074 outpatient sites). Veterans who enroll in the VHA gain access to a comprehensive medical benefit package consisting of preventative and health maintenance care, outpatient and inpatient hospital care, prescriptions, mental health care, home health care, primary care, specialty care, geriatric and extended care, and medical equipment and prosthetics.

### Cohort

A flow chart describing cohort construction is provided in supplementary fig. 7.

Overall, we built a cohort of people with SARS-CoV-2 positive test who survived the first 30 days after the date of the positive test and compared them to a contemporary control group, and separately, to a historical control group where similar cohort selection criteria including surviving the first 30 days of the follow up were applied.

Veterans who used the VHA in 2019 (*n* = 6,244,069) with a positive COVID-19 test between March 1st, 2020 and January 15th, 2021 were enrolled into the COVID-19 cohort (*n* = 169,476). To ensure only post-acute COVID-19 outcomes were examined, we excluded participants who died within 30 days of receiving a positive COVID-19 test result, yielding a cohort of 154,068 participants. The date of the first COVID-19 positive test was set as the start of follow-up, denoted by T_0_; the end of follow-up was set to be the first occurrence of death or January 15th, 2022.

We then built 2 control groups, a contemporary control group of people who lived contemporaneously during the same enrollment period as those in COVID-19 group and, separately, a historical control group from a pre-pandemic era.

The contemporary control cohort initially consisted of veterans who used the VHA in 2019 (*n* = 6,244,069). Those alive by March 1st, 2020 (*n* = 5,963,205) and were not already in the COVID-19 cohort were further enrolled into the contemporary control cohort (*n* = 5,809,137). To ensure a similar distribution of follow-up between the COVID-19 and contemporary control, the start of follow-up for participants in the contemporary control was randomly assigned following the same distribution as participants receiving their first positive COVID-19 test result in the COVID-19 group. Out of the 5,660,999 participants alive at the beginning of follow-up, 5,638,795 were alive 30 days after the beginning of follow-up and were selected as the contemporary control cohort. Follow-up concluded on the first occurrence of death or January 15th, 2022.

We also built a historical control group consisting of 6,463,487 individuals who used the VHA in 2017. Out of those alive on March 1st, 2018 (*n* = 6,152,185), 6,009,794 participants who were not already part of the COVID-19 cohort were enrolled into the historical control. T_0_ was randomly assigned in the historical group using the same follow-up distribution as the COVID-19 group minus 2 years (730 d). In total, out of the 5,876,880 participants who were alive at T_0_, 5,859,621 were alive 30 days after T_0_ and were subsequently selected into the historical control group. Follow-up concluded on the first occurrence of death or January 15th, 2020.

### Data sources

Electronic health records from the VA Corporate Data Warehouse (CDW) were used in this study. Patient demographic information was obtained from the CDW Patient Domain. Outpatient and inpatient clinical information were obtained from the CDW Outpatient Encounters domain and CDW Inpatient Encounters domains, respectively. Medication prescriptions and fillings were obtained from the CDW Outpatient Pharmacy and CDW Bar Code Medication Administration domains. Laboratory test data was collected from the CDW Laboratory Results domain and the COVID-19 Shared Data Resource domain provided information relevant to COVID-19. Additionally, we used the Area Deprivation Index (ADI), defined as a summary measure of income, education, employment, and housing, as a composite variable of contextual factors present at each participants’ residential location^45^.

### Pre-specified outcomes

Pre-specified outcomes were selected based on our prior work on the systematic characterization of Long COVID^1,17,22^ and from evidence in prior literature^8,11,46–50^. Each gastrointestinal outcome was defined based on a corresponding international classification of diseases, 10th revision (ICD10) diagnostic codes^1,16,17,19,51^ or from laboratory test results. Additionally, individual outcomes were also aggregated into a related composite outcome (for example, coagulation outcome consisted of abnormally elevated PT, PTT, and INR). Furthermore, we specified a composite of any gastrointestinal outcome as the first incident occurrence of any of the predefined gastrointestinal outcomes examined in this study (including those based on diagnostic codes or laboratory tests). Incident individual and composite gastrointestinal outcomes during the post-acute phase of COVID-19 were assessed during the follow-up period between the 30 days after T_0_ until the end of follow-up in participants without any history of the outcome in the year prior to T_0_. In instances where the occurrence of outcome may result from medication use (e.g., PT, PTT, INR), the incident outcome was ascertained in participants without history of the related outcome and without exposure to the medications that may affect it in the year prior to T_0_.

### Covariates

We utilized a two pronged approach to covariate selection: 1) covariates were selected based on prior knowledge^1,4,6,7,9,10,16–20,24,26,44,51,52^, 2) in recognition that our knowledge of COVID-19 is evolving, we also employed an algorithmic approach to identify covariates in data domains consisting of diagnoses, medications and laboratory test results. Pre-defined and algorithmically selected covariates were used in modeling and were assessed in the year prior to T_0_.

Pre-defined covariates consisted of age, race (white, black, and other), sex, ADI, body mass index, smoking status (current, former, and never), and measures of healthcare utilization (number of outpatient encounters as well as long-term care utilization^1,16,18^). Additionally, several comorbidities including cancer, cardiovascular disease, chronic kidney disease, chronic lung disease, diabetes, and hypertension were used as pre-defined covariates. Laboratory values consisting of estimated glomerular filtration rate, systolic, and diastolic pressure were also used as pre-defined covariates. Continuous variables were transformed into restricted cubic spline functions to account for possible non-linear relationships.

To supplement our pre-defined covariates, we utilized algorithmically selected covariates from high dimensional data domains consisting of diagnoses, medications, and laboratory test results^53^. Data from patient encounter, prescription, and laboratory domains collected in the year prior to T_0_ were organized into 540 diagnostic groups, 543 medication types, and 62 laboratory test abnormalities. From these three domains (diagnoses, medications, and laboratory test results) we selected variables which occurred in at least 100 participants within each exposure group in acknowledgment of the fact that exceedingly rare variables (those that occurred in fewer than 100 participants in these cohorts) may not substantially influence the examined associations. Univariate relative risks between each variable and exposure was estimated and 100 variables with the highest relative risks were selected for use in statistical analyses^54^. The algorithmic selection process described above was used to independently select high dimensional covariates in each comparison (for example, the COVID-19 vs contemporary control and the COVID-19 vs historical control analyses to assess incident GERD).

### Statistical analysis

Baseline characteristics of the COVID-19, contemporary, and historical control groups were described, and the standardized mean differences between COVID-19 and contemporary control, and between COVID-19 and historical control were calculated.

To estimate the risk of each incident gastrointestinal outcome, we first constructed a sub-cohort of participants without a history of the outcome of interest (for example, the risk of incident GERD was estimated within a sub-cohort of participants without any history of GERD) in the year prior to cohort enrollment.

Within each sub-cohort, three logistic regressions were built to estimate the probabilities of belonging to the target population of VHA users in 2019 (equivalent to the combination of the COVID-19 group and the contemporary control group) for the COVID-19, contemporary, and historical control groups. These probabilities were estimated based on pre-defined and comparison specific algorithmically selected high-dimensional variables and ultimately used as the propensity score. The propensity score was then used to calculate the inverse probability weight (propensity score/(1-propensity score)). To account for the influence of extreme weights and the sample size difference between comparison groups, we prespecified our analytic plan to truncate weight greater than 1000. There were no weights larger than 1000 hence no truncation was conducted. Covariate balance was assessed by standardized mean differences after application of weighting.

After application of inverse probability weighting, cause-specific hazard models where death was considered as a competing risk were used to estimate hazard ratios of incident gastrointestinal outcomes between the COVID-19 and contemporary control groups and the COVID-19 and historical control groups. The survival probability at 1-year within each group was used to estimate the burdens per 1000 participants at 1 year of follow-up in the COVID-19 and control groups; the difference of the estimated burdens between the COVID-19 and control groups was used to compute the excess burdens per 1000 participants at 1 year. Additionally, we conducted analyses in subgroups comprised of age, race, sex, obesity, smoking, diabetes, cardiovascular disease, chronic kidney disease, hyperlipidemia, and hypertension.

The association between COVID-19 and the risks of post-acute gastrointestinal outcomes were further examined by stratifying the COVID-19 cohort into mutually exclusive groups determined by each participants’ care setting during the acute phase of COVID-19 (that is, whether participants were non-hospitalized, hospitalized, or admitted to the intensive care unit during the first 30 days of infection). The statistical approach outlined in the previous paragraph was used to estimate inverse probability weights for each care setting group. Cause-specific hazard models utilizing inverse probability weighting were applied, and hazard ratios, burdens, and excess burdens were calculated.

We conducted a comparative analysis of individuals hospitalized with COVID-19 vs those hospitalized with seasonal influenza. Admission to the hospital was ascertained in the first 30 days after a positive test result (for both COVID-19 and seasonal influenza). Comparisons were conducted using weighted cause-specific hazard models.

We further tested the robustness of our study design by conducting multiple sensitivity analysis. (1) we modified our covariate selection by increasing covariate inclusion to 300 high dimensional variables (instead of the 100 high dimensional variables used in the main analysis) when constructing the inverse probability weight; (2) we restricted covariate selection only to pre-defined variables when constructing the inverse probability weight (no algorithmically selected variables were used); and (3) we applied a doubly robust approach, where associations were estimated by applying both covariate adjustment and the inverse probability weights to survival models^55^.

We tested whether our approach would reproduce known associations by testing fatigue as an outcome – considered a cardinal manifestation of Long COVID – as a positive outcome control. Additionally, we used the approach outlined by Lipsitch et al.^56^ to specify and test a set of negative outcome controls where no prior evidence supports the existence of a causal relationship between COVID-19 exposure and the specified negative outcome controls. Lastly, we tested a pair of negative-exposure controls. We hypothesized that exposure to the influenza vaccine on odd- vs even-numbered calendar days between March 1st, 2020 and January 15th, 2021 would not be associated with increased or decreased risks of the gastrointestinal outcomes examined in our analysis. If successful, application of these negative outcome and negative exposure controls might reduce concern about the presence of spurious biases in study design, covariate selection, analytic approach, outcome ascertainment, residual confounding, and other sources of latent biases^56^.

Estimation of variance when applying weightings was achieved through robust sandwich variance estimators. For every analysis, evidence of statistical significance was considered when a 95% confidence interval excluded unity. All analyses were conducted using SAS Enterprise Guide version 8.2 (SAS Institute), and visualization of results was accomplished using R version 4.04.

### Reporting summary

Further information on research design is available in the Nature Portfolio Reporting Summary linked to this article.

## Supplementary information

Supplemental information

Reporting Summary

## Data Availability

The data that support the findings of this study are available from the US Department of Veterans Affairs. VA data are made freely available to researchers behind the VA firewall with an approved VA study protocol. For more information, please visit https://www.virec.research.va.gov or contact the VA Information Resource Center (VIReC) at VIReC@va.gov. The data generated from this study are available in the supplementary materials file or in the source data file. Source data are provided with this paper.

## Source data

Source data

## Code availability

The analytic code is available at https://github.com/yxie618/longGI.
