# Supplementary material for: Long-term gastrointestinal outcomes of COVID-19
Source: Nat Commun. 2023 Mar 7;14:983. doi: 10.1038/s41467-023-36223-7 (PMC9992516; doi:10.1038/s41467-023-36223-7)
Supplement: Supplementary file 1 — Supplemental information [file 41467_2023_36223_MOESM1_ESM.pdf]

Long-term Gastrointestinal Outcomes of COVID-19

Table of Contents	Page
**Supplementary Figures**	
Supplementary figure 1. Standardized mean difference of predefined and algorithmically selected high dimensional variables	3
Supplementary figure 2. Risks and 1-year burdens of incident post-acute COVID-19 gastrointestinal outcomes compared with the historical control cohort	4
Supplementary figure 3. Risks and 1-year burdens of incident post-acute COVID-19 composite gastrointestinal outcomes compared with the historical control cohort	5
Supplementary figure 4. Subgroup analyses of the risks of incident post-acute CsOVID-19 composite gastrointestinal outcomes compared with the historical control cohort	6
Supplementary figure 5. Risks and 1-year burdens of incident post-acute COVID-19 gastrointestinal outcomes by care setting of the acute infection compared with the historical control cohort	7
Supplementary figure 6. Risks and 1-year burdens of incident post-acute COVID-19 composite gastrointestinal outcomes by care setting of the acute infection compared with the historical control cohort	8
Supplementary figure 7. Cohort contraction flowchart	9
**Supplementary Tables**	
Supplementary table 1. Demographic and health characteristics of COVID-19, contemporary and historical cohorts before weighting	10-11
Supplementary table 2. Demographic and health characteristics of COVID-19, contemporary and historical cohorts after weighting	12-13
Supplementary table 3. Risks and 12-month burdens of post-acute COVID-19 gastrointestinal outcomes compared to contemporary control	14-15
Supplementary table 4. Subgroup analyses of the risks of incident post-acute COVID-19 composite gastrointestinal outcomes compared to contemporary control	16-17
Supplementary table 5. Demographic and health characteristics of the COVID-19 and contemporary cohorts by care setting of the acute infection before weighting	18-20
Supplementary table 6. Demographic and health characteristics of the COVID-19 and contemporary cohorts by care setting of the acute infection after weighting	21-23
Supplementary table 7. Risks and 12-month burdens of post-acute COVID-19 gastrointestinal outcomes by care setting of the acute infection compared to contemporary control	24-27
Supplementary table 8. Demographic and health characteristics of the COVID-19 and historical cohorts by care setting of the acute infection before weighting	28-30
Supplementary table 9. Demographic and health characteristics of the COVID-19 and historical cohorts by care setting of the acute infection after weighting	31-32
Supplementary table 10. Risks and 12-month burdens of post-acute COVID-19 gastrointestinal outcomes compared to historical control	34-35

Supplementary table 11. Subgroup analyses of the risks of incident post-acute COVID-19 composite gastrointestinal outcomes compared to historical control	36-37
Supplementary table 12. Risks and 12-month burdens of post-acute COVID-19 gastrointestinal outcomes by care setting of the acute infection compared to historical control	38-41
Supplementary table 13. Risks of incident post-acute COVID-19 composite gastrointestinal outcomes in comparisons involving participants hospitalized for COVID-19 vs participants hospitalized for seasonal influenza.	42
Supplementary table 14. Sensitivity analysis for any gastrointestinal outcome compared to contemporary and historical controls	43
Supplementary table 15. Sensitivity analysis for any gastrointestinal outcome by care setting of the acute infection compared to contemporary and historical controls	44
Supplementary table 16. Positive and negative outcome controls	45
Supplementary table 17. Negative exposure control: risks and 12-month burdens of gastrointestinal outcomes of those vaccinated for influenza on even-numbered days compared to those vaccinated on odd-numbered days	46-47

Supplementary figure 1. Standardized mean difference of predefined and algorithmically selected high dimensional variables. (a) between COVID-19 and contemporary control; (b) by care setting of the acute infection between COVID-19 and contemporary control; (c) between COVID-19 and historical control; (d) by care setting of the acute infection between COVID-19 and historical control.

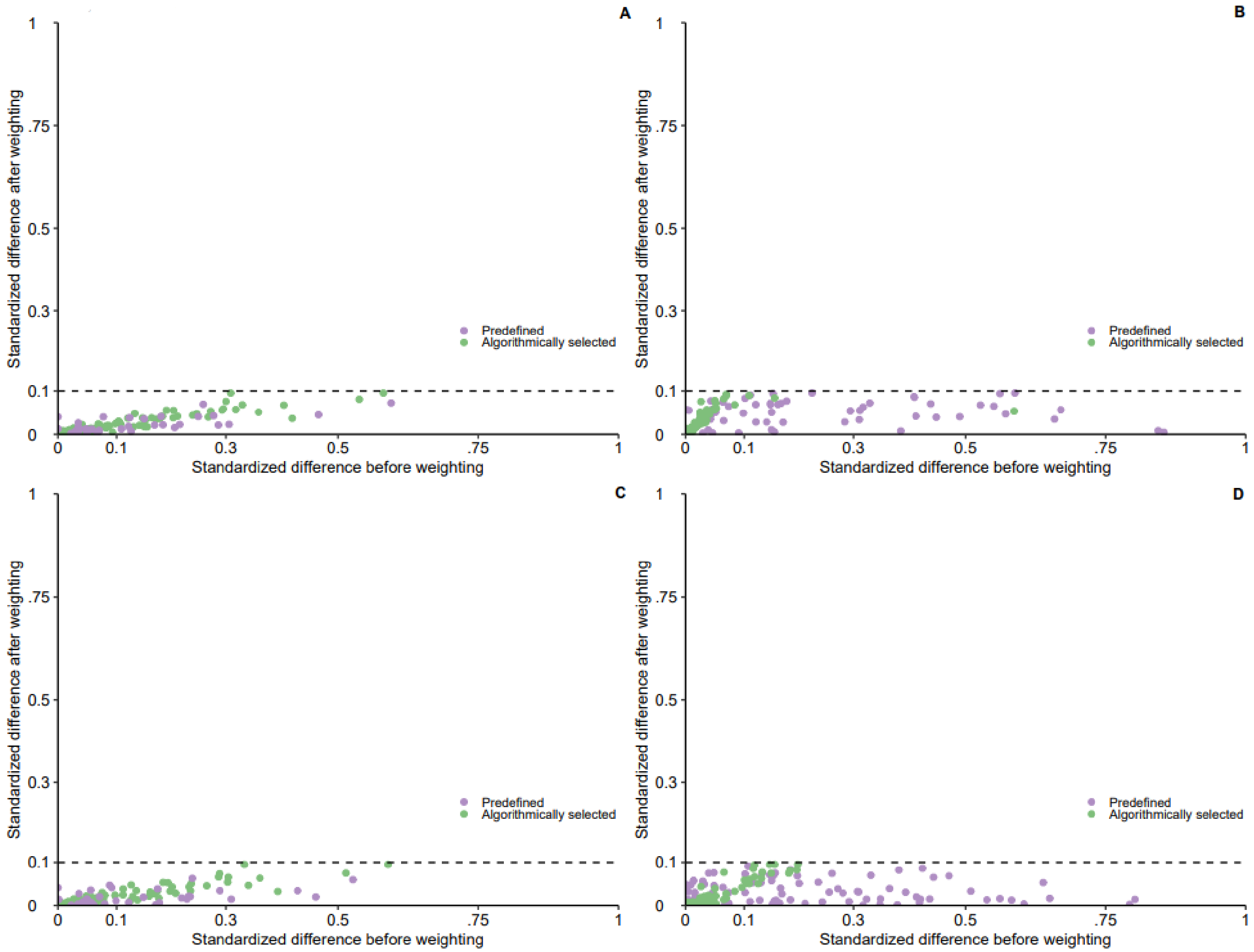

Supplementary figure 2. Risks and 1-year burdens of incident post-acute COVID-19 gastrointestinal outcomes compared with the historical control cohort. Outcomes were ascertained 30 d after the COVID-19-positive test until the end of follow-up. COVID-19 cohort (*n* = 154,068) and historical control cohort (*n* = 5,859,621). Panel A describes the risks and burdens of incident diagnoses (light green) and panel B describes the risks and burdens of incident laboratory abnormalities (orange) (orange)Adjusted HRs (dots) and 95% (error bars) CIs are presented, as are estimated excess burdens (bars) and 95% CIs (error bars). Burdens are presented per 1,000 persons at 12 months of follow up. The dashed line marks a HR of 1.00; lower limits of 95% CIs with values greater than 1.00 indicate significantly increased risk. GERD, gastroesophageal reflux disorder; IBS, irritable bowel syndrome; PT, prothrombin time; PTT, partial thromboplastin time; INR, international normalized ratio; ALT, alanine transaminase; AST, aspartate transaminase; LDH, lactate dehydrogenase; CRP, c-reactive peptide; ALP, alkaline phosphatase; GGT, γ-glutamyl transferase.

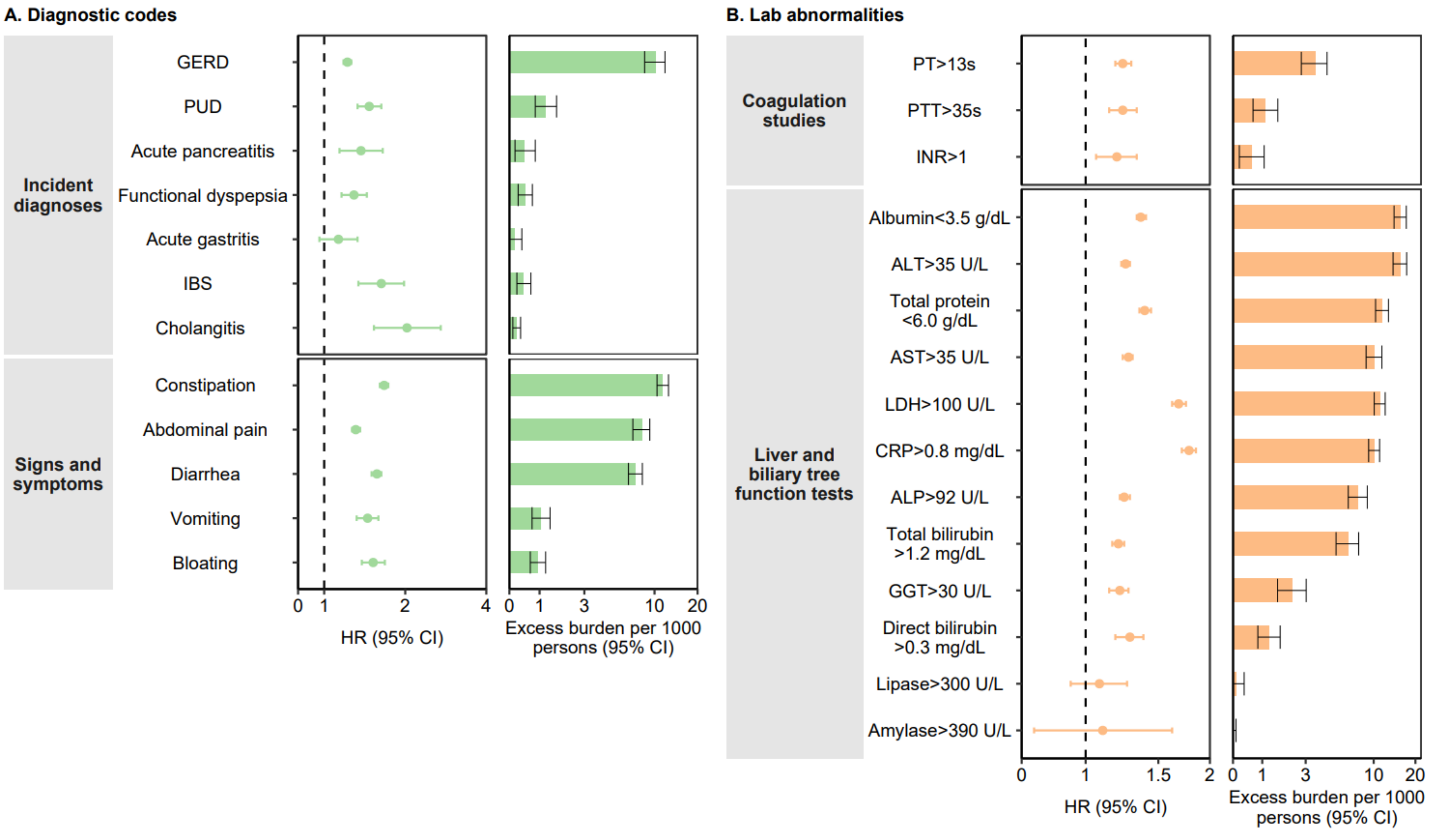

Supplementary figure 3. Risks and 1-year burdens of incident post-acute COVID-19 composite gastrointestinal outcomes compared with the historical control cohort. Composite outcomes consisted of incident diagnoses (GERD, PUD, acute pancreatitis, functional dyspepsia, acute gastritis, IBS, and cholangitis), signs and symptoms (constipation, abdominal pain, diarrhea, vomiting, and bloating), coagulation studies (PT, PTT, INR), liver and biliary tree function tests (albumin, ALT, total protein, AST, LDH, CRP, ALP, total bilirubin, GGT, direct bilirubin, lipase, and amylase) and any gastrointestinal outcome (incident occurrence of any gastrointestinal outcome studied). Outcomes were ascertained 30d after the COVID-19-positive test until the end of follow-up. COVID-19 cohort (*n* = 154,068) and historical control cohort (*n* = 5,859,621). Panel A describes the risks and burdens of incident diagnoses (light green) and panel B describes the risks and burdens of incident laboratory abnormalities (orange). Adjusted HRs (dots) and 95% (error bars) CIs are presented, as are estimated excess burdens (bars) and 95% CIs (error bars). Burdens are presented per 1,000 persons at 12 months of follow up. The dashed line marks a HR of 1.00; lower limits of 95% CIs with values greater than 1.00 indicate significantly increased risk. GERD, gastroesophageal reflux disorder; IBS, irritable bowel syndrome; PT, prothrombin time; PTT, partial thromboplastin time; INR, international normalized ratio; ALT, alanine transaminase; AST, aspartate transaminase; LDH, lactate dehydrogenase; CRP, c-reactive peptide; ALP, alkaline phosphatase; GGT, γ-glutamyl transferase.

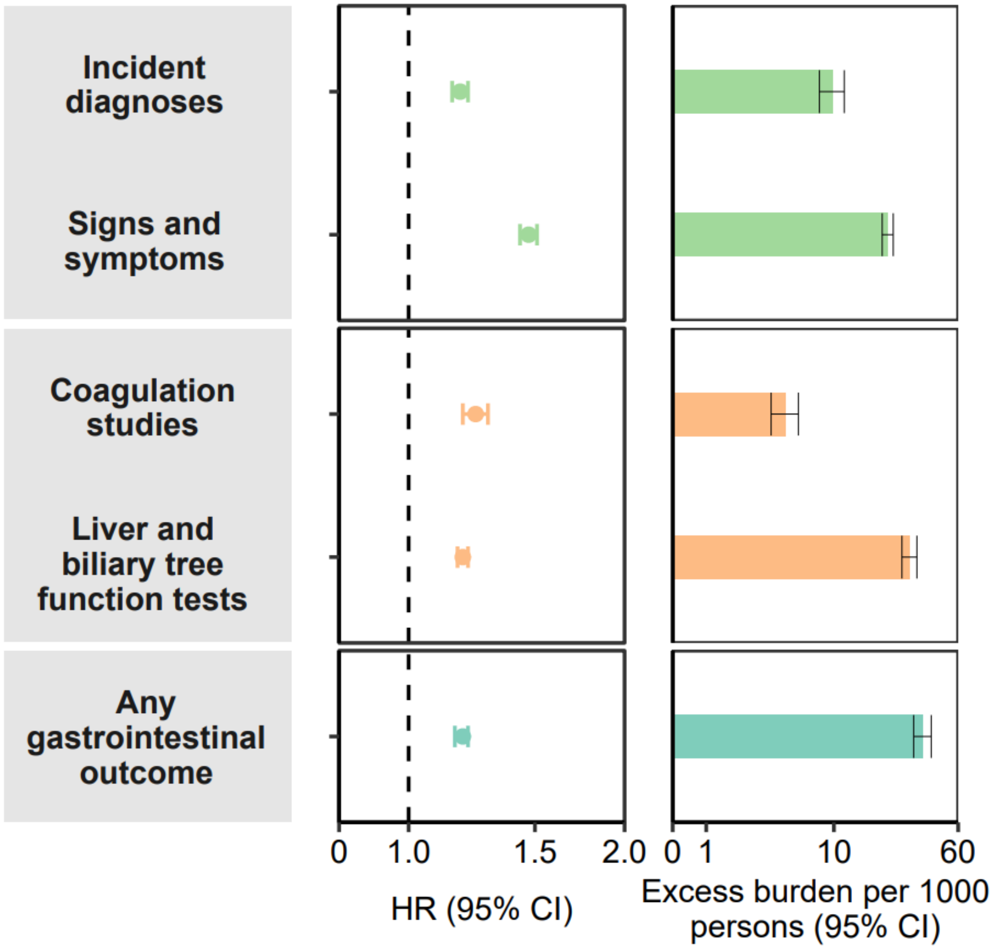

Supplementary figure 4. Subgroup analyses of the risks of incident post-acute COVID-19 composite gastrointestinal outcomes compared with the historical control cohort. Composite outcomes consisted of incident diagnoses (GERD, PUD, acute pancreatitis, functional dyspepsia, acute gastritis, IBS, and cholangitis), signs and symptoms (constipation, abdominal pain, diarrhea, vomiting, and bloating), coagulation studies (PT, PTT, INR), liver and biliary tree function tests (albumin, ALT, total protein, AST, LDH, CRP, ALP, total bilirubin, GGT, direct bilirubin, lipase, and amylase) and any gastrointestinal outcome (incident occurrence of any gastrointestinal outcome studied). Outcomes were ascertained 30d after the COVID-19-positive test until the end of follow-up. COVID-19 cohort (n = 154,068) and historical control cohort (n = 5,859,621). Adjusted HRs (dots) and 95% (error bars) CIs are presented. The dashed line marks a HR of 1.00; lower limits of 95% CIs with values greater than 1.00 indicate significantly increased risk. GERD, gastroesophageal reflux disorder; IBS, irritable bowel syndrome; PT, prothrombin time; PTT, partial thromboplastin time; INR, international normalized ratio; ALT, alanine transaminase; AST, aspartate transaminase; LDH, lactate dehydrogenase; CRP, c-reactive peptide; ALP, alkaline phosphatase; GGT, γ-glutamyl transferase.

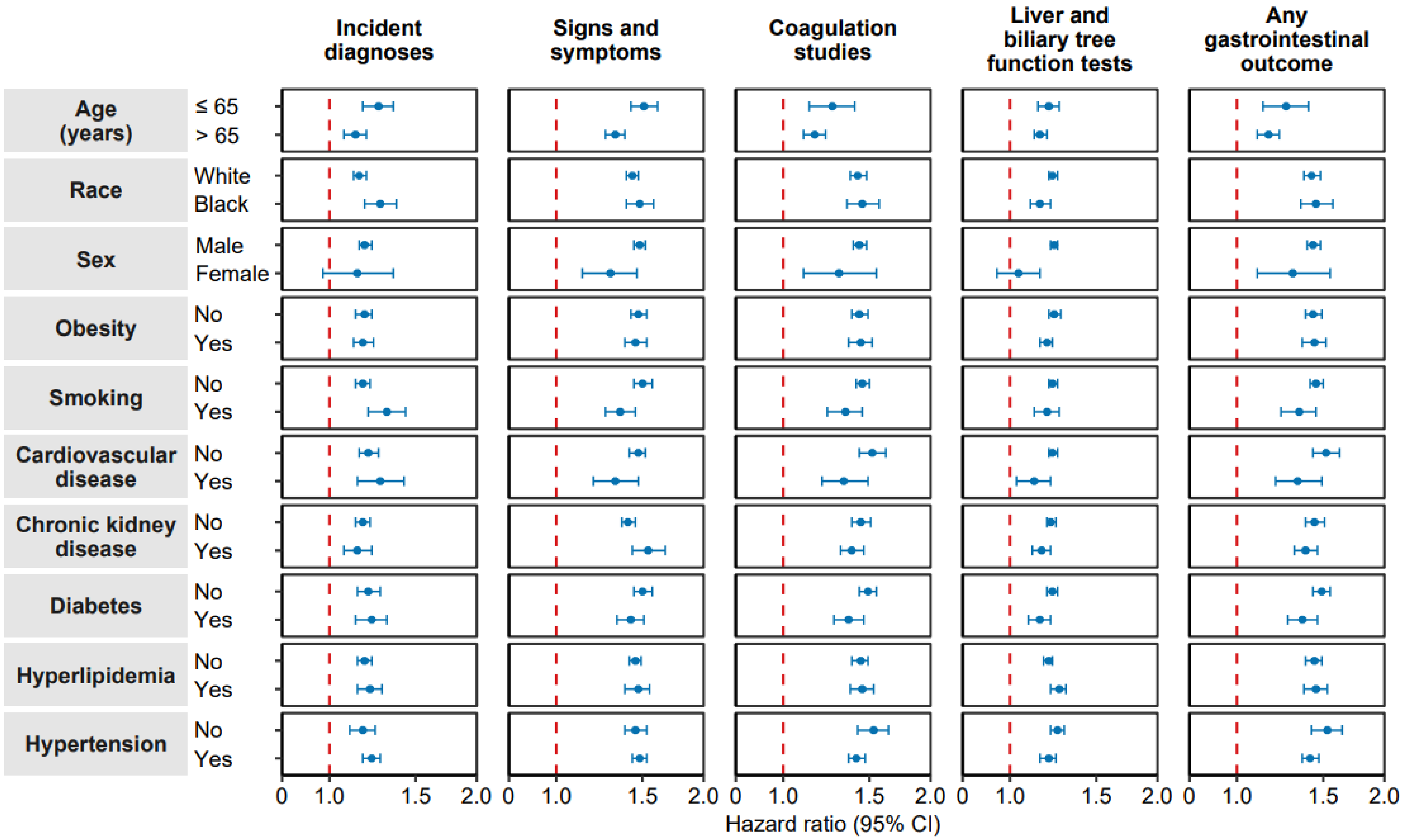

Supplementary figure 5. Risks and 1-year burdens of incident post-acute COVID-19 gastrointestinal outcomes by care setting of the acute infection compared with the historical control cohort. Risks and burdens were assessed at 1-year in mutually exclusive groups comprising non-hospitalized individuals with COVID-19 (green), individuals hospitalized for COVID-19 (orange) and individuals admitted to intensive care for COVID-19 during the acute phase (first 30 d) of COVID-19 (blue). Outcomes were ascertained 30 d after the COVID-19-positive test until the end of follow-up. The historical control cohort served as the referent category. Within the COVID-19 cohort, non-hospitalized (*n* = 131,915), hospitalized (*n* = 16,764), admitted to intensive care (*n* = 5,389) and historical control cohort (*n* = 5,809,908). Panel A describes the risks and burdens of incident diagnoses and panel B describes the risks and burdens of incident laboratory abnormalities. Adjusted HRs (dots) and 95% (error bars) CIs are presented, as are estimated excess burdens (bars) and 95% CIs (error bars). Burdens are presented per 1,000 persons at 12 months of follow up. The dashed line marks a HR of 1.00; lower limits of 95% CIs with values greater than 1.00 indicate significantly increased risk. GERD, gastroesophageal reflux disorder; IBS, irritable bowel syndrome; PT, prothrombin time; PTT, partial thromboplastin time; INR, international normalized ratio; ALT, alanine transaminase; AST, aspartate transaminase; LDH, lactate dehydrogenase; CRP, c-reactive peptide; ALP, alkaline phosphatase; GGT, γ-glutamyl transferase.

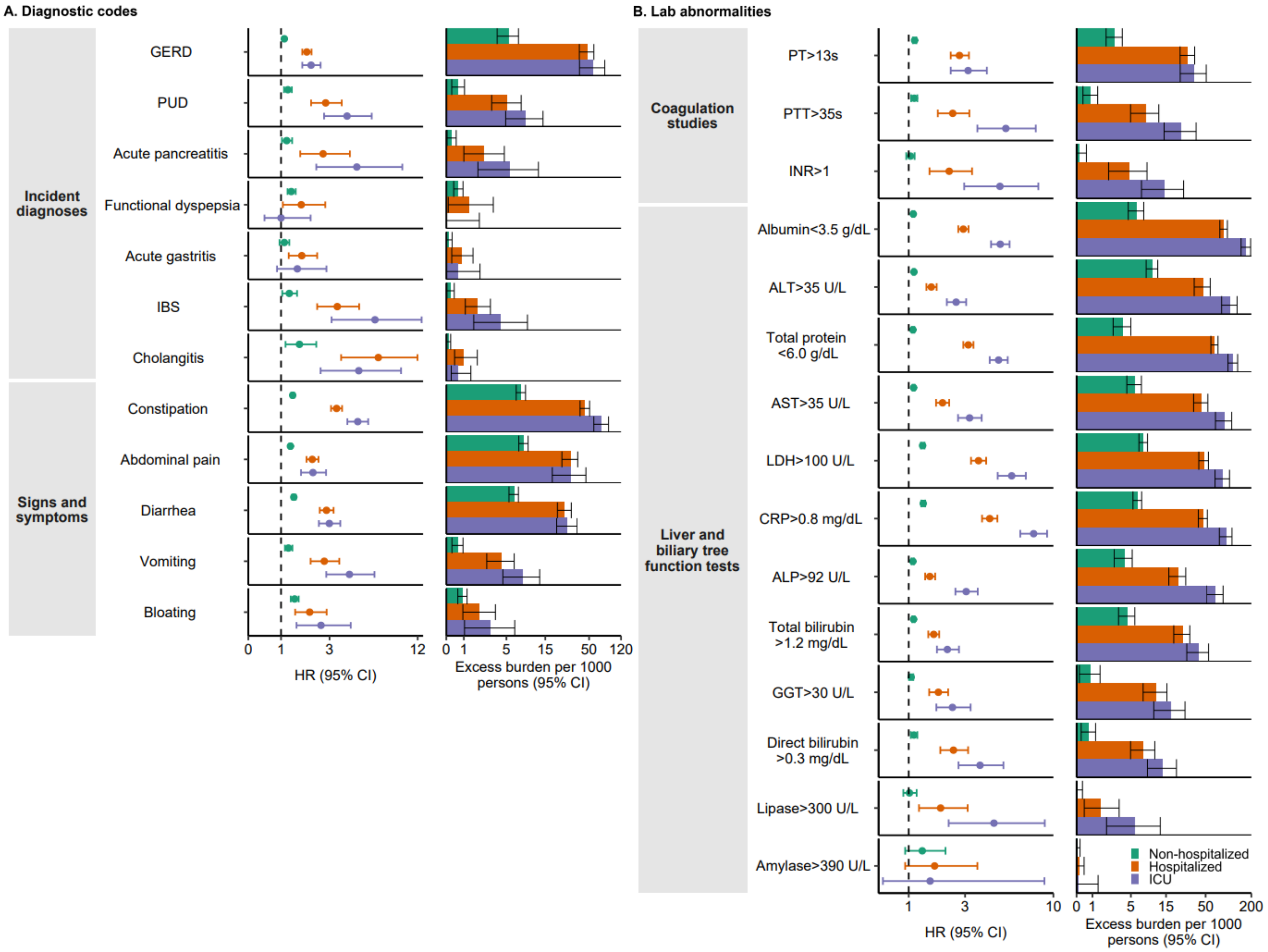

Supplementary figure 6. Risks and 1-year burdens of incident post-acute COVID-19 composite gastrointestinal outcomes by care setting of the acute infection compared with the historical control cohort. Risks and burdens were assessed at 1-year in mutually exclusive groups comprising non-hospitalized individuals with COVID-19 (green), individuals hospitalized for COVID-19 (orange) and individuals admitted to intensive care for COVID-19 during the acute phase (first 30 d) of COVID-19 (blue). Composite outcomes consisted of incident diagnoses (GERD, PUD, acute pancreatitis, functional dyspepsia, acute gastritis, IBS, and cholangitis), signs and symptoms (constipation, abdominal pain, diarrhea, vomiting, and bloating), coagulation studies (PT, PTT, INR), liver and biliary tree function tests (albumin, ALT, total protein, AST, LDH, CRP, ALP, total bilirubin, GGT, direct bilirubin, lipase, and amylase) and any gastrointestinal outcome (incident occurrence of any gastrointestinal outcome studied). Outcomes were ascertained 30 d after the COVID-19-positive test until the end of follow-up. The historical control cohort served as the referent category. Within the COVID-19 cohort, non-hospitalized (*n* = 131,915), hospitalized (*n* = 16,764), admitted to intensive care (*n* = 5,389) and historical control cohort (*n* = 5,809,908). Panel A describes the risks and burdens of incident diagnoses and panel B describes the risks and burdens of incident laboratory abnormalities. Adjusted HRs (dots) and 95% (error bars) CIs are presented, as are estimated excess burdens (bars) and 95% CIs (error bars). Burdens are presented per 1,000 persons at 12 months of follow up. The dashed line marks a HR of 1.00; lower limits of 95% CIs with values greater than 1.00 indicate significantly increased risk. GERD, gastroesophageal reflux disorder; IBS, irritable bowel syndrome; PT, prothrombin time; PTT, partial thromboplastin time; INR, international normalized ratio; ALT, alanine transaminase; AST, aspartate transaminase; LDH, lactate dehydrogenase; CRP, c-reactive peptide; ALP, alkaline phosphatase; GGT, γ-glutamyl transferase.

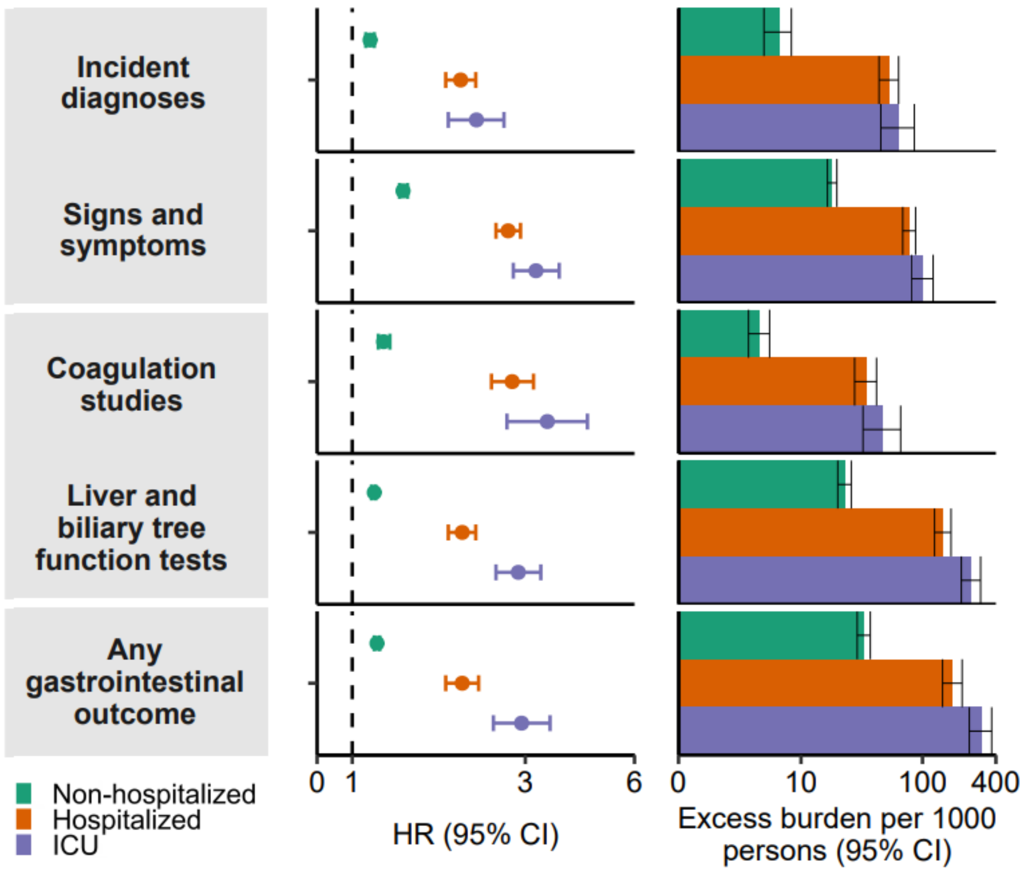

Supplementary figure 7. Cohort construction flowchart

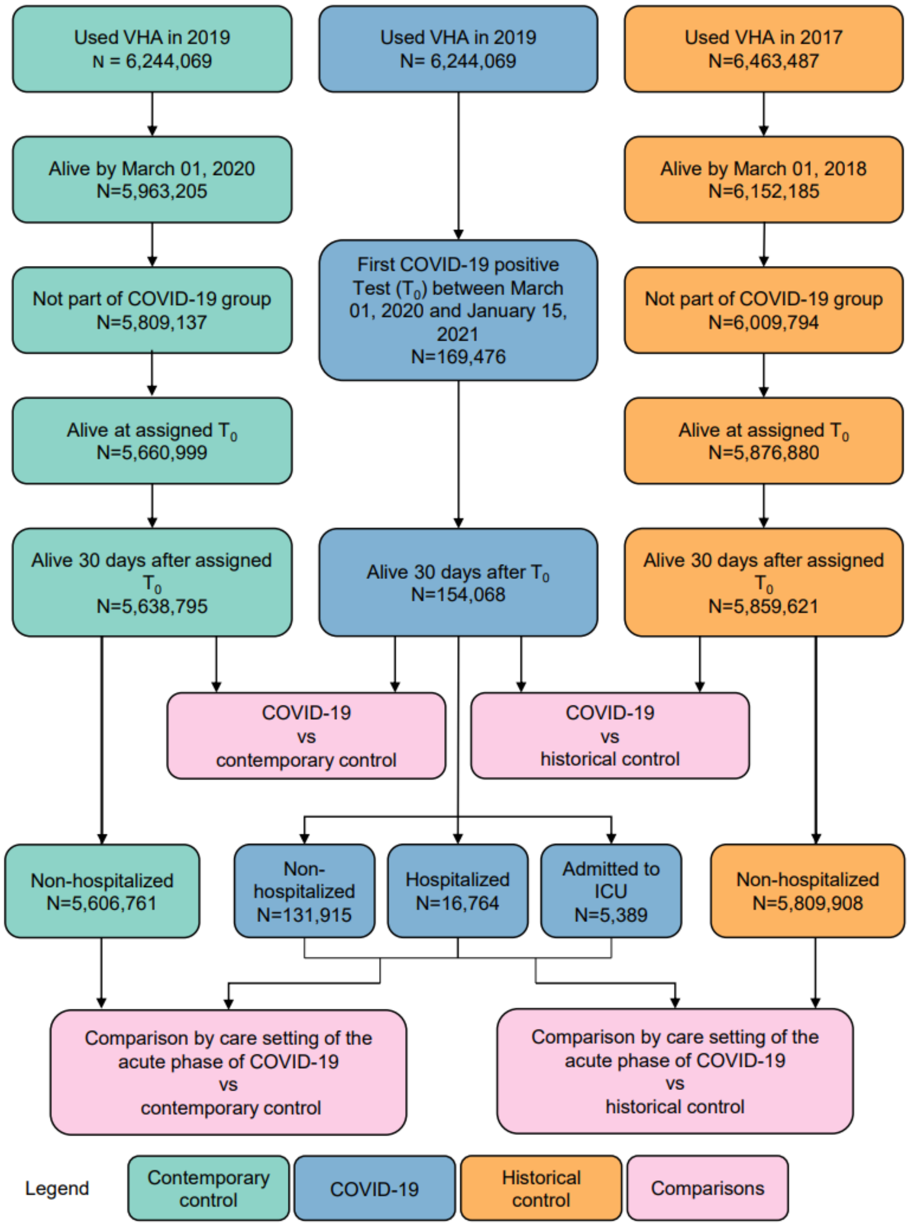

Supplementary table 1. Demographic and health characteristics of COVID-19, contemporary and historical cohorts before weighting

Baseline Characteristics	COVID-19 (N=154,068)	Contemporary control (N=5,638,795)	Historical control (N=5,859,621)	Absolute standardized difference	
				COVID-19 and Contemporary control	COVID-19 and Historical control
**Age, mean (std), yr**	61.42 (15.64)	63.46 (16.23)	62.89 (16.48)	0.13	0.09
**Race, no. (%)**					
White	109,226 (70.89)	4,329,617 (76.78)	4,532,627 (77.35)	0.13	0.15
Black	37,159 (24.12)	1,042,397 (18.49)	1,051,655 (17.95)	0.14	0.15
Other	7,683 (4.99)	266,781 (4.73)	275,339 (4.70)	0.01	0.01
**Sex, no. (%)**					
Male	137,179 (89.04)	5,092,461 (90.31)	5,308,943 (90.60)	0.04	0.05
Female	16,889 (10.96)	546,334 (9.69)	550,678 (9.40)	0.04	0.05
**BMI category, no. (%), kg/m^2**					
<25	21,456 (13.93)	1,062,237 (18.84)	1,123,533 (19.17)	0.13	0.14
25-29.9	49,702 (32.26)	2,190,851 (38.85)	2,332,370 (39.80)	0.14	0.16
≥30	82,910 (53.81)	2,385,707 (42.31)	2,403,718 (41.02)	0.23	0.26
**Smoking status, no. (%)**					
Never	69,208 (44.92)	2,397,935 (42.53)	3,084,196 (52.63)	0.05	0.15
Former	61,229 (39.74)	2,032,158 (36.04)	1,441,928 (24.61)	0.08	0.33
Current	23,631 (15.34)	1,208,702 (21.44)	1,333,497 (22.76)	0.16	0.19
**Area Deprivation Index[a], mean (std)**	55.35 (18.57)	54.67 (19.03)	54.65 (19.12)	0.04	0.04
Clinical Characteristics					
**Outpatient encounter*, no. (%)**					
Zero	4,174 (2.71)	591,209 (10.48)	551,102 (9.41)	0.32	0.28
One	26,585 (17.26)	1,853,280 (32.87)	1,868,338 (31.89)	0.37	0.34
Two or more	123,309 (80.04)	3,194,306 (56.65)	3,440,181 (58.71)	0.52	0.48
**Long-term care, no. (%)**	6,115 (3.97)	33,593 (0.60)	47,598 (0.81)	0.23	0.21

Baseline Characteristics	COVID-19 (N=154,068)	Contemporary control (N=5,638,795)	Historical control (N=5,859,621)	Absolute standardized difference	
				COVID-19 and Contemporary control	COVID-19 and Historical control
**eGFR, mean (std), ml/min/1.73m^2**	77.75 (22.50)	78.59 (20.15)	79.32 (19.95)	0.04	0.07
**Systolic blood pressure, mean (std), mmHg**	132.65 (11.74)	132.65 (12.29)	132.63 (12.63)	0.00	0.00
**Diastolic blood pressure, mean (std), mmHg**	78.32 (7.41)	77.78 (7.54)	77.53 (7.91)	0.07	0.10
**Comorbidities, no. (%)**					
**Cancer**	12,545 (8.14)	346,041 (6.14)	347,533 (5.93)	0.08	0.09
**Cardiovascular disease**	26,677 (17.32)	714,034 (12.66)	764,346 (13.04)	0.13	0.12
**Cerebrovascular disease**	2,176 (1.41)	47,190 (0.84)	53,028 (0.91)	0.05	0.05
**Chronic kidney disease**	30,054 (19.51)	939,892 (16.67)	901,162 (15.38)	0.07	0.11
**Chronic lung disease**	23,521 (15.27)	612,513 (10.86)	655,923 (11.19)	0.13	0.12
**Diabetes mellitus type 2**	49,488 (32.12)	1,279,055 (22.68)	1,338,894 (22.85)	0.21	0.21
**Hyperlipidemia**	95,644 (62.08)	2,767,584 (49.08)	3,009,723 (51.36)	0.26	0.22
**Hypertension**	41,142 (26.70)	1,484,451 (26.33)	1,539,549 (26.27)	0.01	0.01
**Charlson comorbidity index, mean (std)**	1.57 (2.19)	1.02 (1.62)	1.11 (1.74)	0.29	0.23
**History of gastrointestinal disorders, no. (%)**					
**Acute gastritis**	778 (0.51)	14,318 (0.25)	16,641 (0.28)	0.04	0.04
**Acute pancreatitis**	902 (0.59)	17,284 (0.31)	17,781 (0.30)	0.04	0.04
**Cholangitis**	133 (0.09)	2,232 (0.04)	2,279 (0.04)	0.02	0.02
**Functional dyspepsia**	1,227 (0.80)	25,457 (0.45)	28,306 (0.48)	0.04	0.04
**GERD**	44,302 (28.75)	1,210,343 (21.46)	1,190,790 (20.32)	0.17	0.20
**IBS**	382 (0.25)	7,770 (0.14)	7,339 (0.13)	0.03	0.03
**PUD**	1,609 (1.04)	34586 (0.61)	37,130 (0.63)	0.05	0.05

[a]Area Deprivation Index is a measure of socioeconomic disadvantage, with a range from low to high disadvantage of 0 to 100.
*.Data collected within one year before cohort enrollment
std, standard deviation; BMI, body mass index; eGFR, estimated glomerular filtration rate; GERD, gastroesophageal reflux disease; IBS, irritable bowel syndrome; PUD, peptic ulcer disease.

Supplementary table 2. Demographic and health characteristics of COVID-19, contemporary and historical cohorts after weighting

Baseline Characteristics	COVID-19 (N=154,068)	Contemporary control (N=5,638,795)	Historical control (N=5,859,621)	Absolute standardized difference	
				COVID-19 and Contemporary control	COVID-19 and Historical control
**Age, mean (std), yr**	61.75 (16.33)	62.41 (16.42)	62.56 (16.50)	0.04	0.05
**Race, no. (%)**					
White	116,878 (75.86)	4,320,952 (76.63)	4,485,130 (76.54)	0.02	0.02
Black	29,778 (19.33)	1,050,677 (18.63)	1,094,343 (18.68)	0.02	0.02
Other	7,412 (4.81)	267,166 (4.74)	280,148 (4.78)	0.00	0.00
**Sex, no. (%)**					
Male	137,630 (89.33)	5,090,535 (90.28)	5,286,433 (90.22)	0.03	0.03
Female	16,438 (10.67)	548,260 (9.72)	573,188 (9.78)	0.03	0.03
**BMI category, no. (%), kg/m^2**					
<25	28,626 (18.58)	1,055,413 (18.72)	1,095,222 (18.69)	0.00	0.00
25-29.9	55,612 (36.10)	2,181,593 (38.69)	2,287,479 (39.04)	0.05	0.06
≥30	69,830 (45.32)	2,401,788 (42.59)	2,476,862 (42.27)	0.06	0.06
**Smoking status, no. (%)**					
Never	62,578 (40.62)	2,401,281 (42.59)	2,500,300 (42.67)	0.04	0.04
Former	57,213 (37.14)	2,037,691 (36.14)	2,112,100 (36.05)	0.02	0.02
Current	34,276 (22.25)	1,199,823 (21.28)	1,247,279 (21.29)	0.02	0.02
**Area Deprivation Index[a], mean (std)**	55.14 (18.93)	54.61 (19.05)	54.58 (19.05)	0.03	0.03
Clinical Characteristics					
**Outpatient encounter*, no. (%)**					
Zero	14,698 (9.54)	579,668 (10.28)	599,263 (10.23)	0.02	0.02
One	45,937 (29.82)	1,829,225 (32.44)	1,912,639 (32.64)	0.06	0.06
Two or more	93,433 (60.64)	3,229,902 (57.28)	3,347,660 (57.13)	0.07	0.07
**Long-term care, no. (%)**	1,867 (1.21)	38,682 (0.69)	40,549 (0.69)	0.05	0.05

Baseline Characteristics	COVID-19 (N=154,068)	Contemporary control (N=5,638,795)	Historical control (N=5,859,621)	Absolute standardized difference	
				COVID-19 and Contemporary control	COVID-19 and Historical control
**eGFR, mean (std), ml/min/1.73m^2**	79.97 (19.92)	79.72 (19.57)	79.53 (19.77)	0.01	0.02
**Systolic blood pressure, mean (std), mmHg**	132.39 (12.41)	132.55 (12.29)	132.58 (12.32)	0.01	0.02
**Diastolic blood pressure, mean (std), mmHg**	78.06 (7.60)	77.99 (7.54)	77.97 (7.56)	0.01	0.01
**Comorbidities, no. (%)**					
**Cancer**	10,175 (6.60)	349,098 (6.19)	358,667 (6.12)	0.02	0.02
**Cardiovascular disease**	21,505 (13.96)	721,089 (12.79)	742,648 (12.67)	0.03	0.04
**Cerebrovascular disease**	1,451 (0.94)	48,043 (0.85)	49,045 (0.84)	0.01	0.01
**Chronic kidney disease**	26,775 (17.38)	944,103 (16.74)	978,557 (16.70)	0.02	0.02
**Chronic lung disease**	19,329 (12.55)	619,252 (10.98)	634,597 (10.83)	0.05	0.05
**Diabetes mellitus type 2**	36,958 (23.99)	1,293,145 (22.93)	1,337,927 (22.83)	0.02	0.03
**Hyperlipidemia**	79,872 (51.84)	2,787,031 (49.43)	2,892,895 (49.37)	0.05	0.05
**Hypertension**	40,779 (26.47)	1,484,977 (26.34)	1,540,612 (26.29)	0.00	0.00
**Charlson comorbidity index, mean (std)**	0.84 (1.43)	0.87 (1.45)	0.87 (1.41)	0.02	0.02
**History of gastrointestinal disorders, no. (%)**					
**Acute gastritis**	521 (0.34)	14,717 (0.26)	15,294 (0.26)	0.01	0.01
**Acute pancreatitis**	619 (0.40)	17,706 (0.31)	18,516 (0.32)	0.01	0.01
**Cholangitis**	72 (0.05)	2,312 (0.04)	2,402 (0.04)	0.00	0.00
**Functional dyspepsia**	861 (0.56)	25,995 (0.46)	27,130 (0.46)	0.01	0.01
**GERD**	36,494 (23.69)	1,221,363 (21.66)	1,267,846 (21.64)	0.05	0.05
**IBS**	297 (0.19)	7,951 (0.14)	8,203 (0.14)	0.01	0.01
**PUD**	1,160 (0.75)	35,242 (0.63)	36,330 (0.62)	0.02	0.02

[a]Area Deprivation Index is a measure of socioeconomic disadvantage, with a range from low to high disadvantage of 0 to 100.
*.Data collected within one year before cohort enrollment
†888 (0.57%) of the COVID-19 cohort and 28,258 (0.50%) of the contemporary cohort were vaccinated for COVID-19 prior to the start of follow-up
std, standard deviation; BMI, body mass index; eGFR, estimated glomerular filtration rate; GERD, gastroesophageal reflux disease; IBS, irritable bowel syndrome; PUD, peptic ulcer disease.

Supplementary table 3. Risks and 12-month burdens of post-acute COVID-19 gastrointestinal outcomes compared to contemporary control

Outcome*	Hazard Ratio (95% CI)†	COVID-19 burden per 1000 persons at 12 months (95% CI) †	Contemporary control burden per 1000 persons at 12 months (95% CI) †	Absolute burden difference per 1000 persons at 12 months (95% CI) †
	COVID-19 vs Contemporary control			
**Incident diagnoses**	1.37 (1.33, 1.41)	66.11 (64.36, 67.91)	48.74 (48.54, 48.94)	17.37 (15.62, 19.17)
GERD	1.35 (1.31, 1.39)	60.90 (59.23, 62.61)	45.40 (45.21, 45.59)	15.50 (13.83, 17.21)
PUD	1.62 (1.46, 1.79)	4.11 (3.72, 4.54)	2.54 (2.50, 2.58)	1.57 (1.18, 2.00)
Acute pancreatitis	1.46 (1.23, 1.75)	1.89 (1.58, 2.26)	1.29 (1.26, 1.32)	0.60 (0.29, 0.97)
Functional dyspepsia	1.36 (1.22, 1.51)	2.42 (2.18, 2.69)	1.79 (1.75, 1.82)	0.63 (0.39, 0.90)
Acute gastritis	1.47 (1.25, 1.72)	1.49 (1.27, 1.74)	1.01 (0.99, 1.04)	0.47 (0.26, 0.73)
IBS	1.54 (1.28, 1.86)	1.25 (1.04, 1.50)	0.81 (0.79, 0.83)	0.44 (0.22, 0.69)
Cholangitis	2.02 (1.55, 2.63)	0.43 (0.33, 0.56)	0.21 (0.20, 0.23)	0.22 (0.12, 0.35)
**Signs and symptoms**	1.54 (1.50, 1.58)	70.39 (68.68, 72.15)	46.37 (46.19, 46.55)	24.02 (22.30, 25.78)
Constipation	1.60 (1.54, 1.66)	30.67 (29.59, 31.79)	19.33 (19.22, 19.44)	11.34 (10.27, 12.46)
Abdominal pain	1.44 (1.40, 1.49)	34.63 (33.52, 35.77)	24.10 (23.97, 24.23)	10.53 (9.42, 11.67)
Diarrhea	1.58 (1.52, 1.65)	21.88 (21.01, 22.78)	13.88 (13.79, 13.98)	7.99 (7.13, 8.90)
Vomiting	1.52 (1.38, 1.67)	3.56 (3.25, 3.91)	2.35 (2.31, 2.39)	1.21 (0.90, 1.56)
Bloating	1.46 (1.33, 1.61)	2.93 (2.66, 3.22)	2.01 (1.97, 2.04)	0.92 (0.65, 1.21)
**Coagulation studies**	1.59 (1.52, 1.65)	25.31 (24.31, 26.35)	16.04 (15.93, 16.14)	9.27 (8.27, 10.31)
PT>13s	1.61 (1.54, 1.68)	20.95 (20.05, 21.90)	13.06 (12.96, 13.15)	7.90 (6.99, 8.84)
PTT>35s	1.49 (1.38, 1.61)	6.43 (5.94, 6.96)	4.32 (4.26, 4.37)	2.11 (1.63, 2.64)
INR>1	1.48 (1.33, 1.65)	4.21 (3.78, 4.70)	2.84 (2.80, 2.89)	1.37 (0.93, 1.85)
**Liver and biliary tree function tests**	1.30 (1.28, 1.32)	206.83 (203.64, 210.07)	163.35 (163.01, 163.69)	43.49 (40.30, 46.72)
Albumin<3.5 g/dL	1.50 (1.46, 1.54)	64.13 (62.50, 65.81)	43.23 (43.07, 43.40)	20.90 (19.27, 22.57)
ALT>35 U/L	1.25 (1.23, 1.28)	83.97 (82.18, 85.80)	67.58 (67.36, 67.79)	16.39 (14.60, 18.22)
Total protein<6.0 g/dL	1.50 (1.45, 1.54)	44.38 (43.10, 45.69)	29.84 (29.71, 29.98)	14.54 (13.26, 15.85)
AST>35 U/L	1.27 (1.23, 1.30)	52.07 (50.70, 53.47)	41.38 (41.21, 41.54)	10.69 (9.33, 12.10)
LDH>100 U/L	1.54 (1.48, 1.60)	29.42 (28.37, 30.51)	19.20 (19.08, 19.31)	10.22 (9.17, 11.31)
CRP>0.8 mg/dL	1.63 (1.56, 1.69)	24.69 (23.76, 25.67)	15.27 (15.17, 15.36)	9.43 (8.49, 10.40)
ALP>92 U/L	1.28 (1.25, 1.33)	42.29 (41.02, 43.60)	33.08 (32.93, 33.23)	9.21 (7.94, 10.52)

Outcome*	Hazard Ratio (95% CI)†	COVID-19 burden per 1000 persons at 12 months (95% CI) †	Contemporary control burden per 1000 persons at 12 months (95% CI) †	Absolute burden difference per 1000 persons at 12 months (95% CI) †
	COVID-19 vs Contemporary control			
Total bilirubin>1.2 mg/dL	1.22 (1.18, 1.26)	41.70 (40.42, 43.03)	34.31 (34.16, 34.46)	7.40 (6.11, 8.73)
GGT>30 U/L	1.30 (1.24, 1.37)	14.04 (13.33, 14.78)	10.79 (10.71, 10.87)	3.25 (2.54, 3.99)
Direct bilirubin>0.3 mg/dL	1.30 (1.20, 1.40)	6.29 (5.84, 6.78)	4.86 (4.81, 4.92)	1.43 (0.98, 1.92)
Lipase>300 U/L	1.49 (1.27, 1.74)	1.64 (1.40, 1.92)	1.10 (1.07, 1.13)	0.54 (0.30, 0.82)
Amylase>390 U/L	1.54 (1.05, 2.26)	0.20 (0.13, 0.29)	0.13 (0.12, 0.14)	0.07 (0.01, 0.16)
**Any gastrointestinal outcome**	1.36 (1.34, 1.39)	262.93 (258.42, 267.52)	200.6 (200.16, 201.03)	62.34 (57.82, 66.92)

*. Outcomes were ascertained from day 30 after the initial positive COVID-19 test result until end of follow up

†. Adjustment through inverse probability weighting using predefined and algorithmically selected high-dimensional variables.

CI, confidence interval; GERD, gastroesophageal reflux disease; PUD, peptic ulcer disease; IBS, irritable bowel syndrome; PT, prothrombin time; INR, international normalized ratio; PTT; partial thromboplastin time; ALT, alanine transaminase; AST, aspartate transaminase; LDH, lactose dehydrogenase; CRP, c-reactive peptide; ALP, alkaline phosphatase; GGT, gamma-glutamyl transferase.

Supplementary table 4. Subgroup analyses of the risks of incident post-acute COVID-19 composite gastrointestinal outcomes compared to contemporary control

Risk Factors	Hazard ratio (95% CI)† - COVID-19 vs Contemporary control				
	Incident diagnosis	Signs and symptoms	Coagulation studies	Liver and biliary tree function tests	Any gastrointestinal disorder
**Age**					
≤65	1.49 (1.40, 1.58)	1.54 (1.44, 1.65)	1.59 (1.47, 1.73)	1.27 (1.21, 1.33)	1.35 (1.29, 1.41)
>65	1.32 (1.25, 1.39)	1.44 (1.37, 1.50)	1.39 (1.32, 1.47)	1.25 (1.21, 1.29)	1.34 (1.30, 1.39)
**Race**					
White	1.36 (1.31, 1.40)	1.51 (1.47, 1.56)	1.64 (1.57, 1.70)	1.28 (1.26, 1.31)	1.37 (1.34, 1.40)
Black	1.46 (1.36, 1.57)	1.56 (1.46, 1.67)	1.70 (1.57, 1.82)	1.27 (1.21, 1.33)	1.35 (1.28, 1.43)
**Sex**					
Male	1.38 (1.33, 1.42)	1.54 (1.50, 1.59)	1.65 (1.60, 1.70)	1.29 (1.27, 1.32)	1.37 (1.34, 1.40)
Female	1.30 (1.10, 1.53)	1.37 (1.21, 1.56)	1.55 (1.30, 1.84)	1.10 (1.01, 1.21)	1.24 (1.11, 1.39)
**Obesity†**					
No	1.38 (1.33, 1.44)	1.56 (1.51, 1.61)	1.65 (1.59, 1.72)	1.31 (1.28, 1.35)	1.39 (1.35, 1.43)
Yes	1.34 (1.28, 1.41)	1.52 (1.44, 1.59)	1.62 (1.54, 1.72)	1.23 (1.19, 1.26)	1.31 (1.27, 1.35)
**Smoking**					
No/former	1.35 (1.31, 1.40)	1.57 (1.50, 1.64)	1.65 (1.60, 1.70)	1.27 (1.24, 1.29)	1.34 (1.31, 1.37)
Yes	1.49 (1.36, 1.62)	1.41 (1.31, 1.51)	1.53 (1.41, 1.66)	1.26 (1.19, 1.33)	1.36 (1.27, 1.45)

Risk Factors	Hazard ratio (95% CI)† - COVID-19 vs Contemporary control				
	Incident diagnosis	Signs and symptoms	Coagulation studies	Liver and biliary tree function tests	Any gastrointestinal disorder
**Cardiovascular disease**					
No	1.40 (1.33, 1.47)	1.55 (1.49, 1.61)	1.76 (1.66, 1.88)	1.29 (1.26, 1.32)	1.38 (1.35, 1.41)
Yes	1.36 (1.21, 1.52)	1.44 (1.31, 1.58)	1.52 (1.37, 1.68)	1.19 (1.10, 1.27)	1.17 (1.07, 1.28)
**Chronic kidney disease‡**					
No	1.37 (1.32, 1.41)	1.49 (1.44, 1.53)	1.66 (1.59, 1.74)	1.27 (1.25, 1.30)	1.36 (1.33, 1.39)
Yes	1.29 (1.20, 1.38)	1.54 (1.43, 1.67)	1.56 (1.47, 1.65)	1.23 (1.18, 1.29)	1.26 (1.19, 1.34)
**Diabetes**					
No	1.42 (1.35, 1.49)	1.63 (1.56, 1.70)	1.72 (1.65, 1.79)	1.29 (1.26, 1.32)	1.44 (1.39, 1.49)
Yes	1.29 (1.20, 1.39)	1.37 (1.29, 1.46)	1.54 (1.43, 1.65)	1.19 (1.13, 1.26)	1.26 (1.19, 1.34)
**Hyperlipidemia**					
No	1.37 (1.32, 1.42)	1.52 (1.48, 1.57)	1.67 (1.61, 1.73)	1.27 (1.24, 1.30)	1.36 (1.33, 1.39)
Yes	1.38 (1.30, 1.47)	1.52 (1.43, 1.61)	1.63 (1.55, 1.72)	1.29 (1.24, 1.33)	1.36 (1.31, 1.42)
**Hypertension**					
No	1.38 (1.30, 1.46)	1.58 (1.49, 1.66)	1.80 (1.68, 1.93)	1.33 (1.29, 1.38)	1.42 (1.37, 1.47)
Yes	1.35 (1.30, 1.40)	1.50 (1.44, 1.55)	1.58 (1.52, 1.65)	1.22 (1.17, 1.26)	1.26 (1.22, 1.30)

*. Adjustment through inverse probability weighting using predefined and algorithmically selected high-dimensional variables.
†. Obesity was defined based on baseline BMI>30kg/m2
‡. Chronic kidney disease was defined based on baseline outpatient estimated Glomerular Filtration Rate< 60 mL/min/1.73 m2
CI, confidence interval.

Supplementary table 5. Demographic and health characteristics of the COVID-19 and contemporary cohorts by care setting of the acute infection before weighting

Baseline Characteristics	Non-hospitalized (N=131,915)	Hospitalized (N=16,764)	Admitted to intensive care (N=5,389)	Contemporary control (N=5,606,761)	Absolute standardized difference		
					Non-hospitalized and Contemporary control	Hospitalized and Contemporary control	Admitted to intensive care and Contemporary control
**Age, mean (std), yr**	60.23 (15.72)	68.38 (13.38)	68.94 (12.03)	63.44 (16.24)	0.02	0.01	0.02
**Race, no. (%)**							
White	95,282 (72.23)	10,588 (63.16)	3,359 (62.33)	4,307,114 (76.82)	0.11	0.30	0.32
Black	30,209 (22.90)	5,227 (31.18)	1,720 (31.91)	1,034,447 (18.45)	0.11	0.30	0.31
Other	6,424 (4.87)	949 (5.66)	311 (5.77)	265,200 (4.73)	0.01	0.04	0.05
**Sex, no. (%)**							
Male	116,296 (88.16)	15,780 (94.13)	5,105 (94.73)	5,062,345 (90.29)	0.07	0.14	0.17
Female	15,619 (11.84)	984 (5.87)	284 (5.27)	544,416 (9.71)	0.07	0.14	0.17
**BMI category, no. (%), kg/m^2**							
<25	17,439 (13.22)	3,132 (18.68)	882 (16.36)	1,054,071 (18.80)	0.15	0.00	0.06
25-29.9	42,820 (32.46)	5,234 (31.22)	1,644 (30.51)	2,180,469 (38.89)	0.13	0.16	0.18
≥30	71,656 (54.32)	8,399 (50.10)	2,863 (53.13)	2,372,221 (42.31)	0.24	0.16	0.22
**Smoking status, no. (%)**							
Never	59,916 (45.42)	7,123 (42.49)	2,177 (40.40)	2,388,480 (42.60)	0.06	0.00	0.04
Former	52,001 (39.42)	6,851 (40.87)	2,371 (44.00)	2,020,677 (36.04)	0.07	0.10	0.16
Current	19,998 (15.16)	2,791 (16.65)	841 (15.60)	1,198,165 (21.37)	0.16	0.12	0.15
**Area Deprivation Index[a], mean (std)**	55.65 (18.54)	53.18 (18.70)	54.7 (18.59)	54.68 (19.03)	0.01	0.02	0.06
Clinical Characteristics							
**Outpatient encounter*, no. (%)**							
Zero	3,931 (2.98)	186 (1.11)	62 (1.15)	590,953 (10.54)	0.30	0.41	0.41
One	24,866 (18.85)	1,314 (7.84)	405 (7.51)	1,851,352 (33.02)	0.33	0.66	0.67

Baseline Characteristics	Non-hospitalized (N=131,915)	Hospitalized (N=16,764)	Admitted to intensive care (N=5,389)	Contemporary control (N=5,606,761)	Absolute standardized difference		
					Non-hospitalized and Contemporary control	Hospitalized and Contemporary control	Admitted to intensive care and Contemporary control
**Outpatient encounter*, no. (%)**							
Two or more	103,118 (78.17)	15,264 (91.05)	4,922 (91.34)	3,164,456 (56.44)	0.48	0.86	0.87
**Long-term care, no. (%)**	3,891 (2.95)	1,728 (10.31)	498 (9.24)	30,837 (0.55)	0.18	0.44	0.41
**eGFR, mean (std), ml/min/1.73m^2**	79.47 (21.31)	68.28 (26.13)	65.06 (26.78)	78.63 (20.10)	0.00	0.06	0.08
**Systolic blood pressure, mean (std), mmHg**	132.35 (11.69)	134.38 (11.83)	134.56 (12.14)	132.64 (12.29)	0.01	0.05	0.10
**Diastolic blood pressure, mean (std), mmHg**	78.54 (7.37)	77.06 (7.46)	76.99 (7.73)	77.78 (7.54)	0.00	0.04	0.04
**Comorbidities, no. (%)**							
**Cancer**	9,340 (7.08)	2,414 (14.40)	798 (14.80)	339,770 (6.06)	0.04	0.28	0.29
**Cardiovascular disease**	19,550 (14.82)	5,214 (31.10)	1,909 (35.43)	702,527 (12.53)	0.07	0.46	0.56
**Cerebrovascular disease**	1,425 (1.08)	572 (3.41)	179 (3.32)	45,975 (0.82)	0.03	0.18	0.18
**Chronic kidney disease**	21,977 (16.66)	5,945 (35.46)	2,137 (39.66)	930,162 (16.59)	0.00	0.44	0.53
**Chronic lung disease**	17,782 (13.48)	4,211 (25.12)	1,522 (28.25)	603,287 (10.76)	0.08	0.38	0.45
**Diabetes mellitus type 2**	39,100 (29.64)	7,693 (45.89)	2,691 (49.94)	1,266,007 (22.58)	0.16	0.51	0.59
**Hyperlipidemia**	79,413 (60.20)	12,164 (72.56)	4,070 (75.53)	2,745,070 (48.96)	0.23	0.50	0.57
**Hypertension**	34,140 (25.88)	5,292 (31.57)	1,709 (31.72)	1,474,578 (26.30)	0.01	0.12	0.12
**Charlson comorbidity index, mean (std)**	1.34 (1.96)	2.89 (2.87)	3.07 (2.88)	1.01 (1.61)	0.01	0.01	0.03
**History of gastrointestinal disorders, no. (%)**							
**Acute gastritis**	580 (0.44)	149 (0.89)	53 (0.98)	14,017 (0.25)	0.03	0.09	0.09
**Acute pancreatitis**	567 (0.43)	251 (1.50)	78 (1.45)	16,260 (0.29)	0.02	0.13	0.12
**Cholangitis**	79 (0.06)	42 (0.25)	14 (0.26)	2,243 (0.04)	0.01	0.06	0.06

Baseline Characteristics	Non-hospitalized (N=131,915)	Hospitalized (N=16,764)	Admitted to intensive care (N=5,389)	Contemporary control (N=5,606,761)	Absolute standardized difference		
					Non-hospitalized and Contemporary control	Hospitalized and Contemporary control	Admitted to intensive care and Contemporary control
**History of gastrointestinal disorders, no. (%)**							
**Functional dyspepsia**	1,016 (0.77)	159 (0.95)	46 (0.85)	25,230 (0.45)	0.04	0.06	0.05
**GERD**	36,554 (27.71)	5,832 (34.79)	1,922 (35.67)	1,197,604 (21.36)	0.15	0.30	0.32
**IBS**	224 (0.17)	117 (0.70)	41 (0.76)	7,289 (0.13)	0.01	0.09	0.09
**PUD**	1,095 (0.83)	369 (2.20)	143 (2.65)	33,641 (0.60)	0.03	0.14	0.16

[a]Area Deprivation Index is a measure of socioeconomic disadvantage, with a range from low to high disadvantage of 0 to 100.
*.Data collected within one year before cohort enrollment
std, standard deviation; BMI, body mass index; eGFR, estimated glomerular filtration rate; GERD, gastroesophageal reflux disease; IBS, irritable bowel syndrome; PUD, peptic ulcer disease.

Supplementary table 6. Demographic and health characteristics of the COVID-19 and contemporary cohorts by care setting of the acute infection after weighting

Baseline Characteristics	Non-hospitalized (N=131,915)	Hospitalized (N=16,764)	Admitted to intensive care (N=5,389)	Contemporary control (N=5,606,761)	Absolute standardized difference		
					Non-hospitalized and Contemporary control	Hospitalized and Contemporary control	Admitted to intensive care and Contemporary control
**Age, mean (std), yr**	63.07 (16.27)	63.47 (15.71)	63.67 (15.97)	63.38 (16.23)	0.02	0.01	0.02
**Race, no. (%)**							
White	100,084 (75.87)	12,337 (73.59)	3,946 (73.22)	4,298,143 (76.66)	0.02	0.07	0.08
Black	25,420 (19.27)	3,586 (21.39)	1,072 (19.89)	1,042,858 (18.60)	0.02	0.07	0.03
Other	6,424 (4.87)	840 (5.01)	372 (6.90)	265,760 (4.74)	0.01	0.01	0.09
**Sex, no. (%)**							
Male	118,473 (89.81)	15,321 (91.39)	4,991 (92.62)	5,060,662 (90.26)	0.02	0.04	0.08
Female	13,442 (10.19)	1,443 (8.61)	398 (7.38)	546,099 (9.74)	0.02	0.04	0.08
**BMI category, no. (%), kg/m²**							
<25	24,312 (18.43)	3,142 (18.74)	960 (17.81)	1,047,343 (18.68)	0.01	0.00	0.02
25-29.9	48,096 (36.46)	5,876 (35.05)	1,882 (34.92)	2,171,499 (38.73)	0.05	0.08	0.08
≥30	59,507 (45.11)	7,747 (46.21)	2,547 (47.27)	2,388,480 (42.60)	0.05	0.07	0.09
**Smoking status, no. (%)**							
Never	54,309 (41.17)	6,659 (39.72)	2,300 (42.68)	2,391,844 (42.66)	0.03	0.06	0.00
Former	48,914 (37.08)	6,064 (36.17)	1,895 (35.16)	2,026,283 (36.14)	0.02	0.00	0.02
Current	28,692 (21.75)	4,042 (24.11)	1,194 (22.16)	1,188,633 (21.20)	0.01	0.07	0.02
**Area Deprivation Index[a], mean (std)**	54.98 (18.92)	55.04 (19.26)	55.82 (19.32)	54.7 (19.02)	0.01	0.02	0.06
Clinical Characteristics							
**Outpatient encounter*, no. (%)**							
Zero	13,746 (10.42)	1,596 (9.52)	506 (9.39)	579,739 (10.34)	0.00	0.03	0.03
One	42,305 (32.07)	5,814 (34.68)	2,005 (37.20)	1,827,804 (32.60)	0.01	0.04	0.10

Baseline Characteristics	Non-hospitalized (N=131,915)	Hospitalized (N=16,764)	Admitted to intensive care (N=5,389)	Contemporary control (N=5,606,761)	Absolute standardized difference		
					Non-hospitalized and Contemporary control	Hospitalized and Contemporary control	Admitted to intensive care and Contemporary control
**Outpatient encounter*, no. (%)**							
Two or more	75,864 (57.51)	9,354 (55.80)	2,878 (53.41)	3,199,218 (57.06)	0.01	0.03	0.07
**Long-term care, no. (%)**	1,214 (0.92)	223 (1.33)	81 (1.51)	35,883 (0.64)	0.03	0.07	0.08
**eGFR, mean (std), ml/min/1.73m^2**	78.52 (20.60)	77.33 (21.80)	77 (22.18)	78.61 (20.16)	0.00	0.06	0.08
**Systolic blood pressure, mean (std), mmHg**	132.56 (12.40)	133.22 (12.39)	133.91 (12.34)	132.64 (12.27)	0.01	0.05	0.10
**Diastolic blood pressure, mean (std), mmHg**	77.81 (7.62)	78.11 (7.59)	78.11 (7.69)	77.8 (7.53)	0.00	0.04	0.04
**Comorbidities, no. (%)**							
**Cancer**	8,192 (6.21)	1,088 (6.49)	352 (6.53)	342,573 (6.11)	0.00	0.02	0.02
**Cardiovascular disease**	17,123 (12.98)	2,273 (13.56)	774 (14.37)	708,695 (12.64)	0.01	0.03	0.05
**Cerebrovascular disease**	1,148 (0.87)	171 (1.02)	54 (1.01)	47,097 (0.84)	0.00	0.02	0.02
**Chronic kidney disease**	22,848 (17.32)	3,365 (20.07)	1,056 (19.60)	934,647 (16.67)	0.02	0.09	0.08
**Chronic lung disease**	14,722 (11.16)	1,985 (11.84)	606 (11.24)	609,455 (10.87)	0.01	0.03	0.01
**Diabetes mellitus type 2**	30,789 (23.34)	4,476 (26.70)	1,477 (27.40)	1,279,463 (22.82)	0.01	0.09	0.10
**Hyperlipidemia**	67,277 (51.00)	8,638 (51.53)	2,918 (54.14)	2,764,133 (49.30)	0.03	0.04	0.10
**Hypertension**	35,261 (26.73)	4,875 (29.08)	1,619 (30.04)	1,475,139 (26.31)	0.01	0.06	0.08
**Charlson comorbidity index, mean (std)**	1 (1.65)	1.03 (1.60)	1.07 (1.62)	1.02 (1.62)	0.01	0.01	0.03
**History of gastrointestinal disorders, no. (%)**							
**Acute gastritis**	369 (0.28)	79 (0.47)	20 (0.37)	14,017 (0.25)	0.00	0.04	0.02
**Acute pancreatitis**	449 (0.34)	87 (0.52)	23 (0.43)	16,820 (0.30)	0.01	0.03	0.02
**Cholangitis**	53 (0.04)	12 (0.07)	5 (0.09)	2,243 (0.04)	0.00	0.01	0.02

Baseline Characteristics	Non-hospitalized (N=131,915)	Hospitalized (N=16,764)	Admitted to intensive care (N=5,389)	Contemporary control (N=5,606,761)	Absolute standardized difference		
					Non-hospitalized and Contemporary control	Hospitalized and Contemporary control	Admitted to intensive care and Contemporary control
**History of gastrointestinal disorders, no. (%)**							
**Functional dyspepsia**	660 (0.50)	79 (0.47)	26 (0.49)	25,791 (0.46)	0.01	0.00	0.00
**GERD**	29,549 (22.40)	3,822 (22.80)	1,234 (22.90)	1,208,257 (21.55)	0.02	0.03	0.03
**IBS**	211 (0.16)	42 (0.25)	19 (0.36)	7,849 (0.14)	0.01	0.03	0.05
**PUD**	844 (0.64)	134 (0.80)	35 (0.65)	34,201 (0.61)	0.00	0.02	0.01

[a]Area Deprivation Index is a measure of socioeconomic disadvantage, with a range from low to high disadvantage of 0 to 100.
*.Data collected within one year before cohort enrollment
std, standard deviation; BMI, body mass index; eGFR, estimated glomerular filtration rate; GERD, gastroesophageal reflux disease; IBS, irritable bowel syndrome; PUD, peptic ulcer disease.

Supplementary table 7. Risks and 12-month burdens of post-acute COVID-19 gastrointestinal outcomes by care setting of the acute infection compared to contemporary control

Outcome*	Care setting†	Hazard ratio (95% CI)‡ COVID-19 vs Contemporary control	COVID-19 burden per 1000 persons at 12 months (95% CI)‡	Contemporary control burden per 1000 persons at 12 months (95% CI)‡	Burden difference per 1000 persons at 12 months (95% CI)‡
**Incident diagnoses**	Non-hospitalized	1.30 (1.26, 1.33)	63.59 (61.88, 65.36)	49.39 (49.19, 49.59)	14.2 (12.49, 15.97)
	Hospitalized	2.31 (2.10, 2.54)	110.50 (101.08, 120.74)		61.11 (51.69, 71.35)
	Admitted to intensive care	2.55 (2.14, 3.04)	121.16 (102.59, 142.82)		71.77 (53.2, 93.43)
GERD	Non-hospitalized	1.28 (1.24, 1.32)	58.53 (56.90, 60.21)	46.00 (45.81, 46.19)	12.53 (10.9, 14.21)
	Hospitalized	2.24 (2.04, 2.48)	100.30 (91.38, 110.03)		54.3 (45.38, 64.03)
	Admitted to intensive care	2.45 (2.04, 2.96)	109.16 (91.55, 129.92)		63.16 (45.55, 83.92)
PUD	Non-hospitalized	1.35 (1.21, 1.50)	3.49 (3.14, 3.88)	2.60 (2.56, 2.64)	0.9 (0.54, 1.29)
	Hospitalized	3.07 (2.32, 4.05)	7.96 (6.03, 10.49)		5.36 (3.43, 7.89)
	Admitted to intensive care	4.42 (2.99, 6.56)	11.45 (7.74, 16.92)		8.85 (5.14, 14.32)
Acute pancreatitis	Non-hospitalized	1.28 (1.10, 1.48)	1.74 (1.50, 2.02)	1.37 (1.33, 1.40)	0.38 (0.14, 0.65)
	Hospitalized	2.86 (1.80, 4.53)	3.89 (2.46, 6.17)		2.53 (1.09, 4.8)
	Admitted to intensive care	5.07 (2.51, 10.21)	6.90 (3.43, 13.85)		5.53 (2.06, 12.49)
Functional dyspepsia	Non-hospitalized	1.41 (1.26, 1.56)	2.55 (2.29, 2.84)	1.82 (1.78, 1.85)	0.74 (0.48, 1.02)
	Hospitalized	1.78 (1.10, 2.89)	3.24 (2.00, 5.24)		1.42 (0.18, 3.42)
	Admitted to intensive care	1.04 (0.49, 2.18)	1.88 (0.90, 3.95)		0.07 (-0.92, 2.14)
Acute gastritis	Non-hospitalized	1.42 (1.22, 1.64)	1.27 (1.10, 1.47)	0.90 (0.88, 0.92)	0.37 (0.2, 0.58)
	Hospitalized	2.22 (1.61, 3.06)	2.00 (1.45, 2.75)		1.1 (0.55, 1.85)
	Admitted to intensive care	2.01 (1.11, 3.64)	1.81 (1.00, 3.27)		0.91 (0.1, 2.37)
IBS	Non-hospitalized	1.20 (0.98, 1.46)	1.00 (0.82, 1.22)	0.84 (0.81, 0.86)	0.16 (-0.02, 0.39)
	Hospitalized	3.21 (2.24, 4.60)	2.68 (1.87, 3.84)		1.84 (1.03, 3)
	Admitted to intensive care	5.89 (2.91, 11.91)	4.92 (2.43, 9.91)		4.08 (1.6, 9.08)
Cholangitis	Non-hospitalized	1.73 (1.21, 2.48)	0.29 (0.20, 0.41)	0.17 (0.16, 0.17)	0.12 (0.04, 0.24)
	Hospitalized	6.97 (3.85, 12.60)	1.15 (0.64, 2.08)		0.99 (0.47, 1.91)
	Admitted to intensive care	5.14 (2.68, 9.84)	0.85 (0.44, 1.62)		0.68 (0.28, 1.46)
**Signs and symptoms**	Non-hospitalized	1.44 (1.41, 1.48)	67.56 (65.96, 69.21)	47.32 (47.14, 47.50)	20.24 (18.64, 21.89)
	Hospitalized	2.81 (2.60, 3.04)	127.41 (118.29, 137.18)		80.09 (70.96, 89.86)
	Admitted to intensive care	3.36 (2.90, 3.88)	150.21 (131.25, 171.63)		102.89 (83.92, 124.31)

Outcome*	Care setting†	Hazard ratio (95% CI)‡ COVID-19 vs Contemporary control	COVID-19 burden per 1000 persons at 12 months (95% CI)‡	Contemporary control burden per 1000 persons at 12 months (95% CI)‡	Burden difference per 1000 persons at 12 months (95% CI)‡
Constipation	Non-hospitalized	1.35 (1.30, 1.40)	26.92 (25.94, 27.95)	20.09 (19.97, 20.20)	6.84 (5.85, 7.86)
	Hospitalized	3.25 (2.96, 3.58)	63.89 (58.31, 69.99)		43.81 (38.23, 49.91)
	Admitted to intensive care	4.61 (3.90, 5.44)	89.22 (76.14, 104.42)		69.13 (56.05, 84.34)
Abdominal pain	Non-hospitalized	1.45 (1.40, 1.50)	34.36 (33.30, 35.45)	23.82 (23.70, 23.95)	10.53 (9.47, 11.62)
	Hospitalized	2.39 (2.12, 2.69)	55.94 (49.85, 62.74)		32.11 (26.02, 38.92)
	Admitted to intensive care	2.41 (1.88, 3.10)	56.53 (44.34, 71.95)		32.71 (20.51, 48.13)
Diarrhea	Non-hospitalized	1.45 (1.39, 1.51)	20.38 (19.55, 21.24)	14.09 (13.99, 14.18)	6.29 (5.46, 7.15)
	Hospitalized	2.85 (2.52, 3.23)	39.68 (35.10, 44.84)		25.59 (21.01, 30.75)
	Admitted to intensive care	3.00 (2.49, 3.63)	41.71 (34.65, 50.16)		27.62 (20.57, 36.07)
Vomiting	Non-hospitalized	1.31 (1.18, 1.45)	3.09 (2.78, 3.42)	2.36 (2.32, 2.40)	0.72 (0.42, 1.06)
	Hospitalized	2.85 (2.19, 3.71)	6.72 (5.17, 8.74)		4.36 (2.81, 6.38)
	Admitted to intensive care	4.39 (2.96, 6.52)	10.34 (6.97, 15.31)		7.97 (4.61, 12.95)
Bloating	Non-hospitalized	1.41 (1.28, 1.56)	2.85 (2.58, 3.15)	2.02 (1.98, 2.06)	0.83 (0.56, 1.13)
	Hospitalized	1.98 (1.43, 2.74)	3.99 (2.88, 5.52)		1.97 (0.86, 3.50)
	Admitted to intensive care	2.47 (1.48, 4.13)	4.98 (2.98, 8.31)		2.96 (0.96, 6.29)
**Coagulation studies**	Non-hospitalized	1.51 (1.45, 1.57)	24.48 (23.54, 25.46)	16.32 (16.21, 16.43)	8.16 (7.22, 9.14)
	Hospitalized	3.40 (2.98, 3.89)	54.47 (47.80, 62.06)		38.16 (31.48, 45.74)
	Admitted to intensive care	4.24 (3.28, 5.49)	67.44 (52.58, 86.30)		51.12 (36.26, 69.98)
PT>13s	Non-hospitalized	1.52 (1.45, 1.58)	19.78 (18.95, 20.66)	13.09 (13.00, 13.19)	6.69 (5.85, 7.56)
	Hospitalized	3.56 (3.09, 4.11)	45.89 (39.93, 52.71)		32.79 (26.84, 39.61)
	Admitted to intensive care	4.06 (3.10, 5.33)	52.16 (40.02, 67.87)		39.07 (26.92, 54.78)
PTT>35s	Non-hospitalized	1.39 (1.28, 1.50)	6.36 (5.89, 6.87)	4.59 (4.53, 4.65)	1.77 (1.30, 2.28)
	Hospitalized	2.96 (2.28, 3.84)	13.53 (10.45, 17.50)		8.94 (5.86, 12.91)
	Admitted to intensive care	6.41 (4.31, 9.54)	29.08 (19.64, 42.96)		24.49 (15.05, 38.37)
INR>1	Non-hospitalized	1.30 (1.16, 1.45)	3.76 (3.36, 4.21)	2.90 (2.85, 2.94)	0.87 (0.47, 1.31)
	Hospitalized	2.90 (2.03, 4.14)	8.37 (5.86, 11.95)		5.47 (2.96, 9.05)
	Admitted to intensive care	6.13 (3.68, 10.22)	17.64 (10.62, 29.23)		14.74 (7.72, 26.34)
**Liver and biliary tree function tests**	Non-hospitalized	1.25 (1.23, 1.27)	201.33 (198.40, 204.30)	164.77 (164.43, 165.11)	36.56 (33.63, 39.53)
	Hospitalized	2.19 (2.01, 2.39)	326.10 (303.73, 349.66)		161.33 (138.96, 184.89)
	Admitted to intensive care	3.13 (2.71, 3.61)	430.73 (386.42, 477.88)		265.96 (221.65, 313.11)

Outcome*	Care setting†	Hazard ratio (95% CI)‡ COVID-19 vs Contemporary control	COVID-19 burden per 1000 persons at 12 months (95% CI)‡	Contemporary control burden per 1000 persons at 12 months (95% CI)‡	Burden difference per 1000 persons at 12 months (95% CI)‡
Albumin<3.5 g/dL	Non-hospitalized	1.24 (1.21, 1.28)	54.50 (53.04, 56.00)	44.03 (43.86, 44.20)	10.47 (9.01, 11.97)
	Hospitalized	3.22 (2.98, 3.49)	134.98 (125.39, 145.24)		90.95 (81.36, 101.21)
	Admitted to intensive care	5.47 (4.82, 6.22)	218.38 (194.99, 244.14)		174.35 (150.96, 200.11)
ALT>35 U/L	Non-hospitalized	1.18 (1.16, 1.21)	80.80 (79.07, 82.58)	68.67 (68.46, 68.89)	12.13 (10.39, 13.90)
	Hospitalized	1.75 (1.58, 1.93)	116.87 (106.29, 128.43)		48.20 (37.62, 59.75)
	Admitted to intensive care	2.70 (2.32, 3.15)	174.98 (152.13, 200.84)		106.31 (83.45, 132.17)
Total protein<6.0 g/dL	Non-hospitalized	1.20 (1.16, 1.24)	35.94 (34.81, 37.10)	30.00 (29.87, 30.14)	5.94 (4.81, 7.10)
	Hospitalized	3.37 (3.12, 3.64)	97.61 (90.78, 104.93)		67.61 (60.78, 74.92)
	Admitted to intensive care	5.20 (4.59, 5.88)	146.49 (130.61, 164.12)		116.49 (100.61, 134.11)
AST>35 U/L	Non-hospitalized	1.16 (1.12, 1.19)	47.96 (46.66, 49.29)	41.61 (41.45, 41.78)	6.34 (5.05, 7.67)
	Hospitalized	2.11 (1.89, 2.37)	85.91 (77.00, 95.79)		44.30 (35.39, 54.18)
	Admitted to intensive care	3.26 (2.73, 3.89)	129.46 (109.70, 152.47)		87.85 (68.08, 110.86)
LDH>100 U/L	Non-hospitalized	1.30 (1.25, 1.36)	25.82 (24.83, 26.85)	19.85 (19.73, 19.97)	5.97 (4.98, 7.00)
	Hospitalized	3.38 (3.03, 3.77)	65.56 (59.00, 72.83)		45.71 (39.15, 52.98)
	Admitted to intensive care	5.35 (4.42, 6.46)	101.66 (84.88, 121.54)		81.81 (65.03, 101.70)
CRP>0.8 mg/dL	Non-hospitalized	1.31 (1.26, 1.37)	20.66 (19.82, 21.54)	15.75 (15.65, 15.85)	4.91 (4.07, 5.79)
	Hospitalized	3.94 (3.54, 4.38)	60.59 (54.62, 67.20)		44.84 (38.87, 51.45)
	Admitted to intensive care	7.10 (5.96, 8.46)	106.66 (90.34, 125.72)		90.91 (74.59, 109.97)
ALP>92 U/L	Non-hospitalized	1.17 (1.13, 1.21)	38.53 (37.35, 39.76)	33.11 (32.96, 33.25)	5.43 (4.24, 6.65)
	Hospitalized	1.72 (1.55, 1.90)	56.11 (50.87, 61.88)		23.01 (17.77, 28.77)
	Admitted to intensive care	3.19 (2.69, 3.78)	101.79 (86.56, 119.52)		68.68 (53.45, 86.42)
Total bilirubin>1.2 mg/dL	Non-hospitalized	1.15 (1.11, 1.19)	37.62 (36.45, 38.83)	32.81 (32.67, 32.95)	4.81 (3.64, 6.02)
	Hospitalized	1.79 (1.61, 1.99)	57.83 (52.15, 64.11)		25.02 (19.34, 31.30)
	Admitted to intensive care	2.29 (1.90, 2.76)	73.51 (61.39, 87.91)		40.70 (28.58, 55.10)
GGT>30 U/L	Non-hospitalized	1.18 (1.12, 1.25)	12.91 (12.24, 13.62)	10.92 (10.83, 11.00)	1.99 (1.32, 2.70)
	Hospitalized	2.13 (1.79, 2.54)	23.13 (19.46, 27.48)		12.21 (8.54, 16.56)
	Admitted to intensive care	2.73 (2.06, 3.61)	29.48 (22.37, 38.81)		18.57 (11.45, 27.90)
Direct bilirubin>0.3 mg/dL	Non-hospitalized	1.14 (1.05, 1.23)	5.72 (5.27, 6.21)	5.04 (4.99, 5.10)	0.68 (0.23, 1.17)
	Hospitalized	2.48 (1.98, 3.11)	12.46 (9.94, 15.61)		7.42 (4.90, 10.57)
	Admitted to intensive care	3.71 (2.68, 5.13)	18.57 (13.46, 25.60)		13.53 (8.41, 20.56)

Outcome*	Care setting†	Hazard ratio (95% CI)‡ COVID-19 vs Contemporary control	COVID-19 burden per 1000 persons at 12 months (95% CI)‡	Contemporary control burden per 1000 persons at 12 months (95% CI)‡	Burden difference per 1000 persons at 12 months (95% CI)‡
Lipase>300 U/L	Non-hospitalized	1.38 (1.15, 1.66)	1.63 (1.36, 1.95)	1.18 (1.15, 1.21)	0.45 (0.18, 0.77)
	Hospitalized	2.72 (1.75, 4.21)	3.20 (2.07, 4.96)		2.02 (0.89, 3.78)
	Admitted to intensive care	6.14 (3.12, 12.08)	7.22 (3.68, 14.16)		6.04 (2.50, 12.98)
Amylase>390 U/L	Non-hospitalized	1.85 (1.18, 2.89)	0.25 (0.16, 0.39)	0.13 (0.12, 0.14)	0.11 (0.02, 0.25)
	Hospitalized	2.37 (1.18, 4.76)	0.32 (0.16, 0.64)		0.18 (0.02, 0.50)
	Admitted to intensive care	2.18 (0.40, 11.76)	0.29 (0.05, 1.58)		0.16 (-0.08, 1.44)
**Any gastrointestinal outcome**	Non-hospitalized	1.34 (1.31, 1.36)	262.37 (258.3, 266.48)	203.42 (202.99, 203.86)	58.94 (54.88, 63.06)
	Hospitalized	2.30 (2.07, 2.55)	407.05 (375.18, 440.57)		203.63 (171.75, 237.15)
	Admitted to intensive care	3.35 (2.80, 4.01)	533.50 (471.26, 598.42)		330.08 (267.84, 395.00)

*. Outcomes were ascertained from day 30 after the initial positive COVID-19 test result until end of follow up
†. Based on care received within the first 30 days after a positive COVID-19 test result.
‡. Adjustment through inverse probability weighting using predefined and algorithmically selected high-dimensional variables.
CI, confidence interval; GERD, gastroesophageal reflux disease; PUD, peptic ulcer disease; IBS, irritable bowel syndrome; PT, prothrombin time; INR, international normalized ratio; PTT; partial thromboplastin time; ALT, alanine transaminase; AST, aspartate transaminase; LDH, lactose dehydrogenase; CRP, c-reactive peptide; ALP, alkaline phosphatase; GGT, gamma-glutamyl transferase.

Supplementary table 8. Demographic and health characteristics of the COVID-19 and historical cohorts by care setting of the acute infection before weighting

Baseline Characteristics	Non-hospitalized (N=131,915)	Hospitalized (N=16,764)	Admitted to intensive care (N=5,389)	Historical control (N=5,809,908)	Absolute standardized difference		
					Non-hospitalized and Historical control	Hospitalized and Historical control	Admitted to intensive care and Historical control
**Age, mean (std), yr**	60.23 (15.72)	68.38 (13.38)	68.94 (12.03)	62.87 (16.49)	0.02	0.01	0.02
**Race, no. (%)**							
White	95,282 (72.23)	10,588 (63.16)	3,359 (62.33)	4,496,869 (77.40)	0.12	0.32	0.33
Black	30,209 (22.90)	5,227 (31.18)	1,720 (31.91)	1,039,974 (17.90)	0.12	0.31	0.33
Other	6,424 (4.87)	949 (5.66)	311 (5.77)	273,066 (4.70)	0.01	0.04	0.05
**Sex, no. (%)**							
Male	116,296 (88.16)	15,780 (94.13)	5,105 (94.73)	5,262,615 (90.58)	0.08	0.13	0.16
Female	15,619 (11.84)	984 (5.87)	284 (5.27)	547,293 (9.42)	0.08	0.13	0.16
**BMI category, no. (%), kg/m^2**							
<25	17,439 (13.22)	3,132 (18.68)	882 (16.36)	1,110,854 (19.12)	0.16	0.01	0.07
25-29.9	42,820 (32.46)	5,234 (31.22)	1,644 (30.51)	2,315,829 (39.86)	0.15	0.18	0.20
≥30	71,656 (54.32)	8,399 (50.10)	2,863 (53.13)	2,383,224 (41.02)	0.27	0.18	0.24
**Smoking status, no. (%)**							
Never	59,916 (45.42)	7,123 (42.49)	2,177 (40.40)	3,067,050 (52.79)	0.15	0.21	0.25
Former	52,001 (39.42)	6,851 (40.87)	2,371 (44.00)	1,428,075 (24.58)	0.32	0.35	0.42
Current	19,998 (15.16)	2,791 (16.65)	841 (15.60)	1,314,782 (22.63)	0.19	0.15	0.18
**Area Deprivation Index[a], mean (std)**	55.65 (18.54)	53.18 (18.70)	54.7 (18.59)	54.65 (19.11)	0.02	0.02	0.06
Clinical Characteristics							
**Outpatient encounter*, no. (%)**							
Zero	3,931 (2.98)	186 (1.11)	62 (1.15)	550,779 (9.48)	0.27	0.38	0.38
One	24,866 (18.85)	1,314 (7.84)	405 (7.51)	1,865,561 (32.11)	0.31	0.64	0.65

Baseline Characteristics	Non-hospitalized (N=131,915)	Hospitalized (N=16,764)	Admitted to intensive care (N=5,389)	Historical control (N=5,809,908)	Absolute standardized difference		
					Non-hospitalized and Historical control	Hospitalized and Historical control	Admitted to intensive care and Historical control
**Outpatient encounter*, no. (%)**							
Two or more	103,118 (78.17)	15,264 (91.05)	4,922 (91.34)	3,393,567 (58.41)	0.43	0.81	0.82
**Long-term care, no. (%)**	3,891 (2.95)	1,728 (10.31)	498 (9.24)	42,412 (0.73)	0.17	0.43	0.40
**eGFR, mean (std), ml/min/1.73m^2**	79.47 (21.31)	68.28 (26.13)	65.06 (26.78)	79.36 (19.88)	0.01	0.06	0.08
**Systolic blood pressure, mean (std), mmHg**	132.35 (11.69)	134.38 (11.83)	134.56 (12.14)	132.63 (12.63)	0.00	0.05	0.11
**Diastolic blood pressure, mean (std), mmHg**	78.54 (7.37)	77.06 (7.46)	76.99 (7.73)	77.54 (7.91)	0.00	0.04	0.04
**Comorbidities, no. (%)**							
**Cancer**	9,340 (7.08)	2,414 (14.40)	798 (14.80)	338,718 (5.83)	0.05	0.29	0.30
**Cardiovascular disease**	19,550 (14.82)	5,214 (31.10)	1,909 (35.43)	746,573 (12.85)	0.06	0.45	0.55
**Cerebrovascular disease**	1,425 (1.08)	572 (3.41)	179 (3.32)	51,127 (0.88)	0.02	0.17	0.17
**Chronic kidney disease**	21,977 (16.66)	5,945 (35.46)	2,137 (39.66)	887,754 (15.28)	0.04	0.48	0.57
**Chronic lung disease**	17,782 (13.48)	4,211 (25.12)	1,522 (28.25)	641,414 (11.04)	0.07	0.37	0.44
**Diabetes mellitus type 2**	39,100 (29.64)	7,693 (45.89)	2,691 (49.94)	1,319,430 (22.71)	0.16	0.50	0.59
**Hyperlipidemia**	79,413 (60.20)	12,164 (72.56)	4,070 (75.53)	2,974,673 (51.20)	0.18	0.45	0.52
**Hypertension**	34,140 (25.88)	5,292 (31.57)	1,709 (31.72)	1,525,682 (26.26)	0.01	0.12	0.12
**Charlson comorbidity index, mean (std)**	1.34 (1.96)	2.89 (2.87)	3.07 (2.88)	1.01 (1.61)	0.01	0.01	0.03
**History of gastrointestinal disorders, no. (%)**							
**Acute gastritis**	580 (0.44)	149 (0.89)	53 (0.98)	16,268 (0.28)	0.03	0.08	0.09
**Acute pancreatitis**	567 (0.43)	251 (1.50)	78 (1.45)	16,849 (0.29)	0.02	0.13	0.13
**Cholangitis**	79 (0.06)	42 (0.25)	14 (0.26)	2,324 (0.04)	0.01	0.06	0.06

Baseline Characteristics	Non-hospitalized (N=131,915)	Hospitalized (N=16,764)	Admitted to intensive care (N=5,389)	Historical control (N=5,809,908)	Absolute standardized difference		
					Non-hospitalized and Historical control	Hospitalized and Historical control	Admitted to intensive care and Historical control
**History of gastrointestinal disorders, no. (%)**							
**Functional dyspepsia**	1,016 (0.77)	159 (0.95)	46 (0.85)	27,888 (0.48)	0.04	0.06	0.05
**GERD**	36,554 (27.71)	5,832 (34.79)	1,922 (35.67)	1,172,439 (20.18)	0.18	0.33	0.35
**IBS**	224 (0.17)	117 (0.70)	41 (0.76)	6,972 (0.12)	0.01	0.09	0.10
**PUD**	1,095 (0.83)	369 (2.20)	143 (2.65)	35,440 (0.61)	0.03	0.13	0.16

[a]Area Deprivation Index is a measure of socioeconomic disadvantage, with a range from low to high disadvantage of 0 to 100.
*.Data collected within one year before cohort enrollment
std, standard deviation; BMI, body mass index; eGFR, estimated glomerular filtration rate; GERD, gastroesophageal reflux disease; IBS, irritable bowel syndrome; PUD, peptic ulcer disease.

Supplementary table 9. Demographic and health characteristics of the COVID-19 and historical cohorts by care setting of the acute infection after weighting

Baseline Characteristics	Non-hospitalized (N=131,915)	Hospitalized (N=16,764)	Admitted to intensive care (N=5,389)	Historical control (N=5,809,908)	Absolute standardized difference		
					Non-hospitalized and Historical control	Hospitalized and Historical control	Admitted to intensive care and Historical control
**Age, mean (std), yr**	63.07 (16.27)	63.47 (15.71)	63.67 (15.97)	63.34 (16.30)	0.02	0.01	0.02
**Race, no. (%)**							
White	100,084 (75.87)	12,337 (73.59)	3,946 (73.22)	4,449,228 (76.58)	0.02	0.07	0.08
Black	25,420 (19.27)	3,586 (21.39)	1,072 (19.89)	1,082,967 (18.64)	0.02	0.07	0.03
Other	6,424 (4.87)	840 (5.01)	372 (6.90)	277,714 (4.78)	0.00	0.01	0.09
**Sex, no. (%)**							
Male	118,473 (89.81)	15,321 (91.39)	4,991 (92.62)	5,240,537 (90.20)	0.01	0.04	0.09
Female	13,442 (10.19)	1,443 (8.61)	398 (7.38)	569,371 (9.80)	0.01	0.04	0.09
**BMI category, no. (%), kg/m^2**							
<25	24,312 (18.43)	3,142 (18.74)	960 (17.81)	1,080,062 (18.59)	0.00	0.00	0.02
25-29.9	48,096 (36.46)	5,876 (35.05)	1,882 (34.92)	2,275,160 (39.16)	0.06	0.09	0.09
≥30	59,507 (45.11)	7,747 (46.21)	2,547 (47.27)	2,454,686 (42.25)	0.06	0.08	0.10
**Smoking status, no. (%)**							
Never	54,309 (41.17)	6,659 (39.72)	2,300 (42.68)	2,488,384 (42.83)	0.03	0.06	0.00
Former	48,914 (37.08)	6,064 (36.17)	1,895 (35.16)	2,093,891 (36.04)	0.02	0.00	0.02
Current	28,692 (21.75)	4,042 (24.11)	1,194 (22.16)	1,227,634 (21.13)	0.01	0.07	0.02
**Area Deprivation Index[a], mean (std)**	54.98 (18.92)	55.04 (19.26)	55.82 (19.32)	54.67 (19.03)	0.02	0.02	0.06
Clinical Characteristics							
**Outpatient encounter*, no. (%)**							
Zero	13,746 (10.42)	1,596 (9.52)	506 (9.39)	597,259 (10.28)	0.00	0.03	0.03
One	42,305 (32.07)	5,814 (34.68)	2,005 (37.20)	1,909,136 (32.86)	0.02	0.04	0.09

Baseline Characteristics	Non-hospitalized (N=131,915)	Hospitalized (N=16,764)	Admitted to intensive care (N=5,389)	Historical control (N=5,809,908)	Absolute standardized difference		
					Non-hospitalized and Historical control	Hospitalized and Historical control	Admitted to intensive care and Historical control
**Outpatient encounter*, no. (%)**							
Two or more	75,864 (57.51)	9,354 (55.80)	2,878 (53.41)	3,303,514 (56.86)	0.01	0.02	0.07
**Long-term care, no. (%)**	1,214 (0.92)	223 (1.33)	81 (1.51)	36,602 (0.63)	0.03	0.07	0.09
**eGFR, mean (std), ml/min/1.73m^2**	78.52 (20.60)	77.33 (21.80)	77 (22.18)	78.64 (20.23)	0.01	0.06	0.08
**Systolic blood pressure, mean (std), mmHg**	132.56 (12.40)	133.22 (12.39)	133.91 (12.34)	132.61 (12.32)	0.00	0.05	0.11
**Diastolic blood pressure, mean (std), mmHg**	77.81 (7.62)	78.11 (7.59)	78.11 (7.69)	77.81 (7.56)	0.00	0.04	0.04
**Comorbidities, no. (%)**							
**Cancer**	8,192 (6.21)	1,088 (6.49)	352 (6.53)	351,499 (6.05)	0.01	0.02	0.02
**Cardiovascular disease**	17,123 (12.98)	2,273 (13.56)	774 (14.37)	729,143 (12.55)	0.01	0.03	0.05
**Cerebrovascular disease**	1,148 (0.87)	171 (1.02)	54 (1.01)	47,641 (0.82)	0.01	0.02	0.02
**Chronic kidney disease**	22,848 (17.32)	3,365 (20.07)	1,056 (19.60)	965,607 (16.62)	0.02	0.09	0.08
**Chronic lung disease**	14,722 (11.16)	1,985 (11.84)	606 (11.24)	621,079 (10.69)	0.02	0.04	0.02
**Diabetes mellitus type 2**	30,789 (23.34)	4,476 (26.70)	1,477 (27.40)	1,322,335 (22.76)	0.01	0.09	0.10
**Hyperlipidemia**	67,277 (51.00)	8,638 (51.53)	2,918 (54.14)	2,861,380 (49.25)	0.03	0.05	0.10
**Hypertension**	35,261 (26.73)	4,875 (29.08)	1,619 (30.04)	1,526,263 (26.27)	0.01	0.06	0.08
**Charlson comorbidity index, mean (std)**	1.00 (1.65)	1.03 (1.60)	1.07 (1.62)	1.02 (1.60)	0.01	0.01	0.03
**History of gastrointestinal disorders, no. (%)**							
**Acute gastritis**	369 (0.28)	79 (0.47)	20 (0.37)	14,525 (0.25)	0.00	0.04	0.02
**Acute pancreatitis**	449 (0.34)	87 (0.52)	23 (0.43)	17,430 (0.30)	0.01	0.03	0.02
**Cholangitis**	53 (0.04)	12 (0.07)	5 (0.09)	2,324 (0.04)	0.00	0.01	0.02

Baseline Characteristics	Non-hospitalized (N=131,915)	Hospitalized (N=16,764)	Admitted to intensive care (N=5,389)	Historical control (N=5,809,908)	Absolute standardized difference		
					Non-hospitalized and Historical control	Hospitalized and Historical control	Admitted to intensive care and Historical control
**History of gastrointestinal disorders, no. (%)**							
**Functional dyspepsia**	660 (0.50)	79 (0.47)	26 (0.49)	26,726 (0.46)	0.01	0.00	0.00
**GERD**	29,549 (22.40)	3,822 (22.80)	1,234 (22.90)	1,250,292 (21.52)	0.02	0.03	0.03
**IBS**	211 (0.16)	42 (0.25)	19 (0.36)	7,553 (0.13)	0.01	0.03	0.05
**PUD**	844 (0.64)	134 (0.80)	35 (0.65)	34,859 (0.60)	0.00	0.02	0.01

[a]Area Deprivation Index is a measure of socioeconomic disadvantage, with a range from low to high disadvantage of 0 to 100.
*.Data collected within one year before cohort enrollment
std, standard deviation; BMI, body mass index; eGFR, estimated glomerular filtration rate; GERD, gastroesophageal reflux disease; IBS, irritable bowel syndrome; PUD, peptic ulcer disease.

Supplementary table 10. Risks and 12-month burdens of post-acute COVID-19 gastrointestinal outcomes compared to historical control

Outcome*	Hazard Ratio (95% CI)† COVID-19 vs Historical control	COVID-19 burden per 1000 persons at 12 months (95% CI) †	Historical control burden per 1000 persons at 12 months (95% CI) †	Absolute burden difference per 1000 persons at 12 months (95% CI) †
**Incident diagnoses**	1.18 (1.15, 1.21)	66.11 (64.36, 67.91)	56.28 (56.07, 56.49)	9.83 (8.08, 11.63)
GERD	1.17 (1.13, 1.20)	60.90 (59.23, 62.61)	52.48 (52.27, 52.68)	8.42 (6.75, 10.13)
PUD	1.47 (1.33, 1.63)	4.11 (3.72, 4.54)	2.80 (2.75, 2.84)	1.32 (0.92, 1.75)
Acute pancreatitis	1.36 (1.14, 1.62)	1.89 (1.58, 2.26)	1.39 (1.36, 1.42)	0.50 (0.19, 0.87)
Functional dyspepsia	1.31 (1.18, 1.46)	2.42 (2.18, 2.69)	1.85 (1.81, 1.88)	0.57 (0.33, 0.84)
Acute gastritis	1.14 (0.98, 1.34)	1.49 (1.27, 1.74)	1.30 (1.27, 1.33)	0.19 (-0.03, 0.44)
Irritable bowel syndrome	1.65 (1.36, 1.99)	1.25 (1.04, 1.50)	0.76 (0.74, 0.78)	0.49 (0.28, 0.75)
Cholangitis	1.94 (1.49, 2.53)	0.43 (0.33, 0.56)	0.22 (0.21, 0.23)	0.21 (0.11, 0.34)
**Signs and symptoms**	1.47 (1.43, 1.51)	70.39 (68.68, 72.15)	48.54 (48.35, 48.72)	21.86 (20.14, 23.61)
Constipation	1.65 (1.59, 1.71)	30.67 (29.59, 31.79)	18.68 (18.57, 18.79)	11.99 (10.92, 13.11)
Abdominal pain	1.31 (1.27, 1.36)	34.63 (33.52, 35.77)	26.51 (26.37, 26.64)	8.12 (7.01, 9.26)
Diarrhea	1.57 (1.51, 1.63)	21.88 (21.01, 22.78)	14.00 (13.91, 14.1)	7.87 (7.01, 8.78)
Vomiting	1.44 (1.31, 1.58)	3.56 (3.25, 3.91)	2.48 (2.44, 2.52)	1.08 (0.77, 1.43)
Bloating	1.51 (1.38, 1.67)	2.93 (2.66, 3.22)	1.93 (1.90, 1.97)	0.99 (0.73, 1.29)
**Coagulation studies**	1.24 (1.19, 1.29)	25.31 (24.31, 26.35)	20.45 (20.33, 20.57)	4.86 (3.86, 5.90)
PT>13s	1.24 (1.19, 1.30)	20.95 (20.05, 21.90)	16.93 (16.82, 17.04)	4.02 (3.12, 4.97)
PTT>35s	1.24 (1.15, 1.34)	6.43 (5.94, 6.96)	5.18 (5.12, 5.24)	1.25 (0.77, 1.78)
INR>1	1.20 (1.07, 1.34)	4.21 (3.78, 4.70)	3.51 (3.46, 3.57)	0.70 (0.26, 1.18)
**Liver and biliary tree function tests**	1.19 (1.17, 1.21)	206.83 (203.64, 210.07)	176.89 (176.54, 177.24)	29.95 (26.76, 33.18)
Albumin<3.5 g/dL	1.36 (1.33, 1.40)	64.13 (62.50, 65.81)	47.42 (47.25, 47.60)	16.71 (15.08, 18.38)
ALT>35 U/L	1.21 (1.18, 1.24)	83.97 (82.18, 85.80)	69.89 (69.67, 70.11)	14.08 (12.29, 15.91)
Total protein<6.0 g/dL	1.40 (1.36, 1.44)	44.38 (43.10, 45.69)	31.96 (31.82, 32.10)	12.42 (11.15, 13.73)
AST>35 U/L	1.24 (1.21, 1.28)	52.07 (50.70, 53.47)	42.09 (41.93, 42.26)	9.98 (8.61, 11.38)
LDH>100 U/L	1.67 (1.61, 1.73)	29.42 (28.37, 30.51)	17.75 (17.64, 17.86)	11.67 (10.62, 12.76)
CRP>0.8 mg/dL	1.77 (1.70, 1.84)	24.69 (23.76, 25.67)	14.02 (13.93, 14.12)	10.67 (9.74, 11.65)
ALP>92 U/L	1.23 (1.20, 1.27)	42.29 (41.02, 43.60)	34.45 (34.30, 34.59)	7.85 (6.58, 9.16)

Outcome*	Hazard Ratio (95% CI)†	COVID-19 burden per 1000 persons at 12 months (95% CI) †	Historical control burden per 1000 persons at 12 months (95% CI) †	Absolute burden difference per 1000 persons at 12 months (95% CI) †
	COVID-19 vs Historical control			
Total bilirubin>1.2 mg/dL	1.18 (1.14, 1.22)	41.70 (40.42, 43.03)	35.41 (35.26, 35.56)	6.30 (5.01, 7.62)
GGT>30 U/L	1.17 (1.12, 1.24)	14.04 (13.33, 14.78)	11.96 (11.88, 12.05)	2.08 (1.37, 2.82)
Direct bilirubin>0.3 mg/dL	1.31 (1.21, 1.41)	6.29 (5.84, 6.78)	4.82 (4.76, 4.87)	1.48 (1.03, 1.96)
Lipase>300 U/L	1.09 (0.93, 1.28)	1.64 (1.40, 1.92)	1.50 (1.47, 1.53)	0.14 (-0.10, 0.42)
Amylase>390 U/L	1.15 (0.78, 1.68)	0.20 (0.13, 0.29)	0.17 (0.16, 0.18)	0.03 (-0.04, 0.12)
**Any gastrointestinal outcome**	1.19 (1.16, 1.21)	262.93 (258.42, 267.52)	226.76 (226.31, 227.22)	36.17 (31.65, 40.75)

*. Outcomes were ascertained from day 30 after the initial positive COVID-19 test result until end of follow up

†. Adjustment through inverse probability weighting using predefined and algorithmically selected high-dimensional variables.

CI, confidence interval; GERD, gastroesophageal reflux disease; PUD, peptic ulcer disease; IBS, irritable bowel syndrome; PT, prothrombin time; INR, international normalized ratio; PTT; partial thromboplastin time; ALT, alanine transaminase; AST, aspartate transaminase; LDH, lactose dehydrogenase; CRP, c-reactive peptide; ALP, alkaline phosphatase; GGT, gamma-glutamyl transferase.

Supplementary table 11. Subgroup analyses of the risks of incident post-acute COVID-19 composite gastrointestinal outcomes compared to historical control

Risk Factors	Hazard ratio (95% CI)† - COVID-19 vs Historical control				
	Incident diagnosis	Signs and symptoms	Coagulation studies	Liver and biliary tree function tests	Any gastrointestinal disorder
**Age**					
≤65	1.26 (1.17, 1.35)	1.51 (1.42, 1.61)	1.26 (1.13, 1.40)	1.20 (1.14, 1.26)	1.16 (1.10, 1.22)
>65	1.13 (1.07, 1.19)	1.32 (1.26, 1.38)	1.16 (1.10, 1.22)	1.15 (1.12, 1.19)	1.13 (1.09, 1.17)
**Race**					
White	1.15 (1.12, 1.19)	1.43 (1.39, 1.47)	1.42 (1.37, 1.48)	1.22 (1.20, 1.25)	1.19 (1.16, 1.22)
Black	1.27 (1.18, 1.37)	1.48 (1.39, 1.58)	1.45 (1.35, 1.57)	1.15 (1.10, 1.21)	1.15 (1.09, 1.22)
**Sex**					
Male	1.18 (1.15, 1.22)	1.48 (1.44, 1.52)	1.43 (1.39, 1.48)	1.23 (1.21, 1.25)	1.19 (1.17, 1.22)
Female	1.14 (0.97, 1.35)	1.29 (1.13, 1.46)	1.30 (1.10, 1.55)	1.04 (0.94, 1.15)	1.04 (0.93, 1.16)
**Obesity†**					
No	1.18 (1.13, 1.22)	1.47 (1.42, 1.53)	1.43 (1.38, 1.49)	1.23 (1.20, 1.27)	1.20 (1.16, 1.23)
Yes	1.17 (1.12, 1.23)	1.45 (1.38, 1.53)	1.44 (1.36, 1.52)	1.19 (1.15, 1.22)	1.16 (1.12, 1.20)
**Smoking**					
No/former	1.17 (1.13, 1.21)	1.50 (1.44, 1.57)	1.45 (1.41, 1.50)	1.22 (1.20, 1.25)	1.18 (1.16, 1.21)
Yes	1.31 (1.20, 1.43)	1.35 (1.26, 1.45)	1.34 (1.23, 1.45)	1.19 (1.12, 1.26)	1.18 (1.11, 1.26)

Risk Factors	Hazard ratio (95% CI)† - COVID-19 vs Historical control				
	Incident diagnosis	Signs and symptoms	Coagulation studies	Liver and biliary tree function tests	Any gastrointestinal disorder
**Cardiovascular disease**					
No	1.20 (1.15, 1.26)	1.47 (1.41, 1.52)	1.52 (1.43, 1.62)	1.22 (1.20, 1.25)	1.20 (1.17, 1.22)
Yes	1.27 (1.14, 1.42)	1.32 (1.19, 1.47)	1.33 (1.20, 1.49)	1.12 (1.03, 1.21)	1.08 (0.98, 1.18)
**Chronic kidney disease‡**					
No	1.17 (1.13, 1.21)	1.40 (1.36, 1.45)	1.44 (1.38, 1.51)	1.21 (1.19, 1.24)	1.18 (1.15, 1.21)
Yes	1.14 (1.07, 1.22)	1.54 (1.43, 1.67)	1.38 (1.31, 1.46)	1.16 (1.11, 1.21)	1.12 (1.06, 1.19)
**Diabetes**					
No	1.20 (1.14, 1.27)	1.50 (1.44, 1.57)	1.49 (1.43, 1.55)	1.22 (1.19, 1.25)	1.22 (1.17, 1.26)
Yes	1.22 (1.13, 1.31)	1.42 (1.33, 1.51)	1.36 (1.27, 1.46)	1.15 (1.09, 1.21)	1.18 (1.11, 1.26)
**Hyperlipidemia**					
No	1.18 (1.14, 1.22)	1.45 (1.41, 1.49)	1.44 (1.38, 1.49)	1.20 (1.17, 1.22)	1.18 (1.15, 1.21)
Yes	1.21 (1.14, 1.28)	1.47 (1.38, 1.55)	1.45 (1.37, 1.53)	1.26 (1.21, 1.30)	1.20 (1.15, 1.25)
**Hypertension**					
No	1.17 (1.10, 1.24)	1.45 (1.38, 1.53)	1.53 (1.42, 1.64)	1.25 (1.21, 1.29)	1.21 (1.16, 1.25)
Yes	1.22 (1.17, 1.27)	1.48 (1.43, 1.53)	1.41 (1.36, 1.47)	1.20 (1.15, 1.24)	1.17 (1.14, 1.21)

*. Adjustment through inverse probability weighting using predefined and algorithmically selected high-dimensional variables.
†. Obesity was defined based on baseline BMI>30kg/m2
‡. Chronic kidney disease was defined based on baseline outpatient estimated Glomerular Filtration Rate< 60 mL/min/1.73 m2
CI, confidence interval.

Supplementary table 12. Risks and 12-month burdens of post-acute COVID-19 gastrointestinal outcomes by care setting of the acute infection compared to historical control

Outcome*	Care setting†	Hazard ratio (95% CI)‡ COVID-19 vs Historical control	COVID-19 burden per 1000 persons at 12 months (95% CI)‡	Historical control burden per 1000 persons at 12 months (95% CI)‡	Burden difference per 1000 persons at 12 months (95% CI)‡
**Incident diagnoses**	Non-hospitalized	1.12 (1.09, 1.15)	63.59 (61.88, 65.36)	57.06 (56.85, 57.27)	6.54 (4.82, 8.30)
	Hospitalized	1.99 (1.81, 2.19)	110.50 (101.08, 120.74)		53.44 (44.02, 63.68)
	Admitted to intensive care	2.20 (1.84, 2.62)	121.16 (102.59, 142.82)		64.10 (45.53, 85.76)
GERD	Non-hospitalized	1.11 (1.07, 1.14)	58.53 (56.90, 60.21)	53.11 (52.91, 53.32)	5.41 (3.78, 7.09)
	Hospitalized	1.94 (1.76, 2.14)	100.30 (91.38, 110.03)		47.18 (38.27, 56.91)
	Admitted to intensive care	2.12 (1.76, 2.55)	109.16 (91.55, 129.92)		56.05 (38.44, 76.80)
PUD	Non-hospitalized	1.23 (1.10, 1.37)	3.49 (3.14, 3.88)	2.85 (2.80, 2.89)	0.65 (0.30, 1.04)
	Hospitalized	2.80 (2.12, 3.70)	7.96 (6.03, 10.49)		5.11 (3.18, 7.65)
	Admitted to intensive care	4.04 (2.72, 5.98)	11.45 (7.74, 16.92)		8.60 (4.89, 14.07)
Acute pancreatitis	Non-hospitalized	1.19 (1.03, 1.38)	1.74 (1.50, 2.02)	1.46 (1.43, 1.49)	0.28 (0.04, 0.55)
	Hospitalized	2.67 (1.68, 4.23)	3.89 (2.46, 6.17)		2.43 (1.00, 4.70)
	Admitted to intensive care	4.73 (2.35, 9.53)	6.90 (3.43, 13.85)		5.43 (1.96, 12.39)
Functional dyspepsia	Non-hospitalized	1.35 (1.22, 1.51)	2.55 (2.29, 2.84)	1.89 (1.85, 1.92)	0.66 (0.41, 0.95)
	Hospitalized	1.72 (1.06, 2.78)	3.24 (2.00, 5.24)		1.35 (0.11, 3.35)
	Admitted to intensive care	1.00 (0.48, 2.10)	1.88 (0.90, 3.95)		0.00 (-0.99, 2.07)
Acute gastritis	Non-hospitalized	1.11 (0.95, 1.28)	1.27 (1.10, 1.47)	1.15 (1.13, 1.18)	0.12 (-0.05, 0.32)
	Hospitalized	1.74 (1.26, 2.39)	2.00 (1.45, 2.75)		0.85 (0.30, 1.60)
	Admitted to intensive care	1.57 (0.87, 2.84)	1.81 (1.00, 3.27)		0.66 (-0.15, 2.12)
IBS	Non-hospitalized	1.28 (1.04, 1.56)	1.00 (0.82, 1.22)	0.78 (0.76, 0.81)	0.22 (0.03, 0.44)
	Hospitalized	3.43 (2.39, 4.91)	2.68 (1.87, 3.84)		1.90 (1.09, 3.05)
	Admitted to intensive care	6.29 (3.11, 12.72)	4.92 (2.43, 9.91)		4.13 (1.65, 9.13)
Cholangitis	Non-hospitalized	1.65 (1.15, 2.35)	0.29 (0.20, 0.41)	0.17 (0.16, 0.18)	0.11 (0.03, 0.24)
	Hospitalized	6.62 (3.66, 11.96)	1.15 (0.64, 2.08)		0.98 (0.46, 1.91)
	Admitted to intensive care	4.88 (2.55, 9.35)	0.85 (0.44, 1.62)		0.67 (0.27, 1.45)
**Signs and symptoms**	Non-hospitalized	1.38 (1.35, 1.42)	67.56 (65.96, 69.21)	49.39 (49.21, 49.58)	18.17 (16.57, 19.82)
	Hospitalized	2.69 (2.49, 2.91)	127.41 (118.29, 137.18)		78.02 (68.89, 87.79)
	Admitted to intensive care	3.21 (2.78, 3.72)	150.21 (131.25, 171.63)		100.82 (81.85, 122.24)

Outcome*	Care setting[†]	Hazard ratio (95% CI)[‡] COVID-19 vs Historical control	COVID-19 burden per 1000 persons at 12 months (95% CI)[‡]	Historical control burden per 1000 persons at 12 months (95% CI)[‡]	Burden difference per 1000 persons at 12 months (95% CI)[‡]
Constipation	Non-hospitalized	1.40 (1.35, 1.45)	26.92 (25.94, 27.95)	19.30 (19.19, 19.41)	7.63 (6.64, 8.65)
	Hospitalized	3.39 (3.08, 3.72)	63.89 (58.31, 69.99)		44.59 (39.01, 50.69)
	Admitted to intensive care	4.80 (4.06, 5.66)	89.22 (76.14, 104.42)		69.92 (56.84, 85.12)
Abdominal pain	Non-hospitalized	1.32 (1.28, 1.36)	34.36 (33.30, 35.45)	26.15 (26.02, 26.28)	8.21 (7.15, 9.30)
	Hospitalized	2.17 (1.93, 2.45)	55.94 (49.85, 62.74)		29.79 (23.70, 36.6)
	Admitted to intensive care	2.20 (1.71, 2.82)	56.53 (44.34, 71.95)		30.39 (18.19, 45.81)
Diarrhea	Non-hospitalized	1.44 (1.38, 1.51)	20.38 (19.55, 21.24)	14.16 (14.06, 14.26)	6.22 (5.39, 7.08)
	Hospitalized	2.84 (2.51, 3.22)	39.68 (35.10, 44.84)		25.51 (20.94, 30.68)
	Admitted to intensive care	2.99 (2.47, 3.61)	41.71 (34.65, 50.16)		27.55 (20.49, 36.00)
Vomiting	Non-hospitalized	1.25 (1.13, 1.39)	3.09 (2.78, 3.42)	2.47 (2.43, 2.51)	0.62 (0.31, 0.95)
	Hospitalized	2.73 (2.10, 3.55)	6.72 (5.17, 8.74)		4.25 (2.70, 6.27)
	Admitted to intensive care	4.20 (2.83, 6.24)	10.34 (6.97, 15.31)		7.86 (4.50, 12.84)
Bloating	Non-hospitalized	1.47 (1.33, 1.62)	2.85 (2.58, 3.15)	1.94 (1.91, 1.98)	0.91 (0.64, 1.20)
	Hospitalized	2.06 (1.49, 2.85)	3.99 (2.88, 5.52)		2.05 (0.94, 3.58)
	Admitted to intensive care	2.57 (1.54, 4.29)	4.98 (2.98, 8.31)		3.04 (1.04, 6.37)
**Coagulation studies**	Non-hospitalized	1.22 (1.18, 1.27)	24.48 (23.54, 25.46)	20.06 (19.94, 20.18)	4.42 (3.48, 5.40)
	Hospitalized	2.76 (2.42, 3.16)	54.47 (47.80, 62.06)		34.42 (27.74, 42.00)
	Admitted to intensive care	3.45 (2.67, 4.45)	67.44 (52.58, 86.30)		47.38 (32.52, 66.24)
PT>13s	Non-hospitalized	1.17 (1.12, 1.22)	19.78 (18.95, 20.65)	16.91 (16.80, 17.02)	2.87 (2.04, 3.75)
	Hospitalized	2.75 (2.39, 3.18)	45.89 (39.93, 52.71)		28.98 (23.02, 35.80)
	Admitted to intensive care	3.14 (2.39, 4.12)	52.16 (40.01, 67.87)		35.26 (23.11, 50.96)
PTT>35s	Non-hospitalized	1.16 (1.07, 1.25)	6.36 (5.89, 6.87)	5.50 (5.44, 5.56)	0.86 (0.39, 1.37)
	Hospitalized	2.47 (1.91, 3.2)	13.53 (10.45, 17.50)		8.03 (4.95, 12.01)
	Admitted to intensive care	5.35 (3.60, 7.96)	29.08 (19.64, 42.96)		23.58 (14.14, 37.46)
INR>1	Non-hospitalized	1.05 (0.93, 1.17)	3.76 (3.36, 4.21)	3.60 (3.55, 3.65)	0.16 (-0.24, 0.61)
	Hospitalized	2.33 (1.63, 3.33)	8.37 (5.86, 11.95)		4.77 (2.26, 8.35)
	Admitted to intensive care	4.94 (2.96, 8.23)	17.64 (10.62, 29.23)		14.04 (7.02, 25.64)
**Liver and biliary tree function tests**	Non-hospitalized	1.15 (1.13, 1.16)	201.33 (198.40, 204.30)	178.24 (177.89, 178.59)	23.09 (20.16, 26.06)
	Hospitalized	2.01 (1.84, 2.19)	326.10 (303.73, 349.66)		147.86 (125.49, 171.42)
	Admitted to intensive care	2.87 (2.49, 3.31)	430.73 (386.41, 477.89)		252.49 (208.18, 299.64)

Outcome*	Care setting†	Hazard ratio (95% CI)‡ COVID-19 vs Historical control	COVID-19 burden per 1000 persons at 12 months (95% CI)‡	Historical control burden per 1000 persons at 12 months (95% CI)‡	Burden difference per 1000 persons at 12 months (95% CI)‡
Albumin<3.5 g/dL	Non-hospitalized	1.13 (1.10, 1.16)	54.50 (53.04, 56.00)	48.46 (48.28, 48.64)	6.04 (4.58, 7.53)
	Hospitalized	2.92 (2.70, 3.16)	134.98 (125.39, 145.24)		86.52 (76.93, 96.78)
	Admitted to intensive care	4.96 (4.37, 5.63)	218.38 (194.99, 244.14)		169.92 (146.52, 195.67)
ALT>35 U/L	Non-hospitalized	1.15 (1.12, 1.17)	80.80 (79.06, 82.58)	70.94 (70.72, 71.16)	9.87 (8.13, 11.64)
	Hospitalized	1.69 (1.53, 1.87)	116.87 (106.29, 128.43)		45.93 (35.35, 57.49)
	Admitted to intensive care	2.61 (2.24, 3.05)	174.98 (152.13, 200.84)		104.04 (81.19, 129.90)
Total protein<6.0 g/dL	Non-hospitalized	1.12 (1.09, 1.16)	35.94 (34.81, 37.10)	32.11 (31.97, 32.26)	3.83 (2.70, 4.99)
	Hospitalized	3.15 (2.92, 3.40)	97.61 (90.78, 104.93)		65.50 (58.67, 72.81)
	Admitted to intensive care	4.85 (4.29, 5.49)	146.49 (130.61, 164.12)		114.38 (98.50, 132.00)
AST>35 U/L	Non-hospitalized	1.14 (1.11, 1.17)	47.96 (46.66, 49.29)	42.29 (42.13, 42.46)	5.66 (4.37, 7.00)
	Hospitalized	2.08 (1.85, 2.33)	85.91 (77.00, 95.79)		43.62 (34.71, 53.50)
	Admitted to intensive care	3.21 (2.69, 3.83)	129.46 (109.70, 152.47)		87.17 (67.40, 110.18)
LDH>100 U/L	Non-hospitalized	1.41 (1.36, 1.47)	25.82 (24.83, 26.85)	18.36 (18.25, 18.47)	7.46 (6.47, 8.49)
	Hospitalized	3.66 (3.28, 4.08)	65.56 (58.99, 72.83)		47.20 (40.64, 54.47)
	Admitted to intensive care	5.79 (4.79, 6.99)	101.66 (84.88, 121.55)		83.31 (66.52, 103.19)
CRP>0.8 mg/dL	Non-hospitalized	1.43 (1.37, 1.49)	20.66 (19.82, 21.54)	14.47 (14.37, 14.57)	6.19 (5.35, 7.06)
	Hospitalized	4.29 (3.85, 4.77)	60.59 (54.61, 67.20)		46.12 (40.14, 52.72)
	Admitted to intensive care	7.74 (6.49, 9.22)	106.66 (90.34, 125.72)		92.19 (75.86, 111.25)
ALP>92 U/L	Non-hospitalized	1.12 (1.08, 1.15)	38.53 (37.34, 39.76)	34.54 (34.39, 34.69)	3.99 (2.80, 5.22)
	Hospitalized	1.64 (1.49, 1.82)	56.11 (50.87, 61.88)		21.57 (16.33, 27.33)
	Admitted to intensive care	3.05 (2.58, 3.62)	101.79 (86.56, 119.53)		67.25 (52.02, 84.98)
Total bilirubin>1.2 mg/dL	Non-hospitalized	1.14 (1.10, 1.17)	37.62 (36.45, 38.83)	33.16 (33.02, 33.3)	4.46 (3.29, 5.67)
	Hospitalized	1.77 (1.59, 1.96)	57.83 (52.15, 64.11)		24.67 (18.99, 30.95)
	Admitted to intensive care	2.26 (1.88, 2.73)	73.51 (61.39, 87.91)		40.35 (28.23, 54.75)
GGT>30 U/L	Non-hospitalized	1.07 (1.01, 1.13)	12.91 (12.24, 13.62)	12.07 (11.98, 12.16)	0.84 (0.17, 1.55)
	Hospitalized	1.93 (1.62, 2.29)	23.13 (19.46, 27.48)		11.06 (7.39, 15.41)
	Admitted to intensive care	2.46 (1.86, 3.26)	29.48 (22.37, 38.81)		17.42 (10.30, 26.74)
Direct bilirubin>0.3 mg/dL	Non-hospitalized	1.15 (1.06, 1.25)	5.72 (5.27, 6.22)	4.99 (4.94, 5.05)	0.73 (0.28, 1.22)
	Hospitalized	2.50 (2.00, 3.14)	12.46 (9.94, 15.61)		7.47 (4.95, 10.62)
	Admitted to intensive care	3.74 (2.71, 5.18)	18.57 (13.46, 25.60)		13.58 (8.47, 20.61)

Outcome*	Care setting†	Hazard ratio (95% CI)‡ COVID-19 vs Historical control	COVID-19 burden per 1000 persons at 12 months (95% CI)‡	Historical control burden per 1000 persons at 12 months (95% CI)‡	Burden difference per 1000 persons at 12 months (95% CI)‡
Lipase>300 U/L	Non-hospitalized	1.02 (0.85, 1.23)	1.63 (1.36, 1.95)	1.59 (1.56, 1.63)	0.04 (-0.23, 0.36)
	Hospitalized	2.01 (1.30, 3.12)	3.20 (2.07, 4.96)		1.61 (0.47, 3.36)
	Admitted to intensive care	4.55 (2.31, 8.95)	7.22 (3.68, 14.16)		5.63 (2.08, 12.57)
Amylase>390 U/L	Non-hospitalized	1.40 (0.90, 2.19)	0.25 (0.16, 0.39)	0.18 (0.17, 0.19)	0.07 (-0.02, 0.21)
	Hospitalized	1.80 (0.90, 3.60)	0.32 (0.16, 0.64)		0.14 (-0.02, 0.46)
	Admitted to intensive care	1.65 (0.31, 8.91)	0.29 (0.05, 1.57)		0.12 (-0.12, 1.40)
**Any gastrointestinal outcome**	Non-hospitalized	1.17 (1.15, 1.19)	262.37 (258.31, 266.47)	229.16 (228.71, 229.61)	33.21 (29.14, 37.32)
	Hospitalized	2.01 (1.81, 2.23)	407.05 (375.18, 440.58)		177.89 (146.02, 211.41)
	Admitted to intensive care	2.93 (2.45, 3.51)	533.50 (471.24, 598.45)		304.34 (242.10, 369.26)

*. Outcomes were ascertained from day 30 after the initial positive COVID-19 test result until end of follow up

†. Based on care received within the first 30 days after a positive COVID-19 test result.

‡. Adjustment through inverse probability weighting using predefined and algorithmically selected high-dimensional variables.

CI, confidence interval; GERD, gastroesophageal reflux disease; PUD, peptic ulcer disease; IBS, irritable bowel syndrome; PT, prothrombin time; INR, international normalized ratio; PTT; partial thromboplastin time; ALT, alanine transaminase; AST, aspartate transaminase; LDH, lactose dehydrogenase; CRP, c-reactive peptide; ALP, alkaline phosphatase; GGT, gamma-glutamyl transferase.

Supplementary Table 13. Risks of incident post-acute COVID-19 composite gastrointestinal outcomes in comparisons involving participants hospitalized for COVID-19 vs participants hospitalized for seasonal influenza.

Outcome*	Hazard ratio (95% CI)† Hospitalized COVID-19 vs hospitalized influenza
Incident diagnoses	1.60 (0.99, 2.57)
Signs and symptoms	1.22 (0.79, 1.88)
Coagulation studies	3.22 (1.60, 6.49)
Liver and biliary tree function tests	2.54 (1.48, 4.37)
Any gastrointestinal outcome	2.78 (1.36, 5.67)

*. Outcomes were ascertained from 30 days after the initial positive COVID-19 test result until end of follow up.
†. Adjustment through inverse probability weighting using predefined and algorithmically selected high-dimensional variables.
CI, confidence interval.

Supplementary table 14. Sensitivity analysis for any gastrointestinal outcome compared to contemporary and historical controls

Analysis	Any gastrointestinal outcome Hazard ratio (95% CI)*	
	Contemporary	Historical
Used 300 algorithmically selected high dimensional variables	1.36 (1.33, 1.39)	1.18 (1.16, 1.21)
Did not use any high dimensional variables	1.40 (1.37, 1.42)	1.23 (1.21, 1.25)
Doubly robust	1.28 (1.25, 1.30)	1.11 (1.09, 1.14)

*. Outcomes were ascertained from day 30 after the initial positive COVID-19 test result until end of follow up.
CI, confidence interval.

Supplementary table 15. Sensitivity analysis for any gastrointestinal outcome by care setting of the acute infection compared to contemporary and historical controls

Analysis	Care Setting†	Any gastrointestinal outcome Hazard ratio (95% CI)*	
		Contemporary	Historical
Used 300 algorithmically selected high dimensional variables	Non-hospitalized	1.34 (1.31, 1.36)	1.17 (1.15, 1.19)
	Hospitalized	2.30 (2.07, 2.55)	2.00 (1.80, 2.23)
	Admitted to intensive care	3.35 (2.80, 4.01)	2.92 (2.44, 3.50)
Did not use any high dimensional variables	Non-hospitalized	1.34 (1.32, 1.37)	1.18 (1.16, 1.20)
	Hospitalized	2.30 (2.07, 2.56)	2.03 (1.83, 2.25)
	Admitted to intensive care	3.36 (2.81, 4.02)	2.96 (2.47, 3.54)
Doubly robust	Non-hospitalized	1.30 (1.28, 1.33)	1.14 (1.12, 1.16)
	Hospitalized	2.29 (2.07, 2.53)	1.99 (1.80, 2.02)
	Admitted to intensive care	3.31 (2.77, 3.96)	2.89 (2.42, 3.46)

*. Outcomes were ascertained from day 30 after the initial positive COVID-19 test result until end of follow up.
†. Based on care received within the first 30 days after a positive COVID-19 test result.
CI, confidence interval.

Supplementary table 16. Positive and negative outcome controls

Outcome*	Hazard Ratio (95% CI)	
	**COVID-19 vs contemporary control (as the reference)**†	**COVID-19 vs historical control (as the reference)**†
**Positive outcome control**		
Fatigue	1.84 (1.78, 1.88)	1.85 (1.80, 1.91)
**ICD 10 negative outcome controls**		
Melanoma in situ	1.08 (0.91, 1.21)	1.03 (0.90, 1.22)
Lichen planus	1.00 (0.84, 1.23)	0.98 (0.80, 1.19)
Hypertrichosis	0.98 (0.76, 1.26)	0.99 (0.77, 1.25)
**Laboratory negative outcome controls**		
Magnesium<1.5 mg/dL	1.02 (0.83, 1.15)	1.01 (0.87, 1.17)

*. Outcomes were ascertained from day 30 after the initial positive COVID-19 test result until end of follow up

†. Adjustment through inverse probability weighting using predefined and algorithmically selected high-dimensional variables.

CI, confidence interval.

Supplementary table 17. Negative exposure control: risks and 12-month burdens of gastrointestinal outcomes of those vaccinated for influenza on even-numbered days compared to those vaccinated on odd-numbered days

Outcome*	Hazard Ratio (95% CI)†	Influenza vaccination on even days burden per 1000 persons at 12 months (95% CI)†	Influenza vaccination on odd days burden per 1000 persons at 12 months (95% CI)†	Absolute burden difference per 1000 persons at 12 months (95% CI)†
	Influenza vaccination on even days vs odd days			
**Incident diagnoses**	1.00 (0.94, 1.07)	14.32 (13.43, 15.28)	14.25 (13.92, 14.59)	0.07 (-0.82, 1.02)
GERD	1.01 (0.94, 1.08)	13.05 (12.20, 13.95)	12.97 (12.65, 13.28)	0.09 (-0.76, 0.99)
PUD	1.10 (0.88, 1.38)	0.92 (0.74, 1.15)	0.83 (0.77, 0.90)	0.09 (-0.09, 0.31)
Acute pancreatitis	1.10 (0.80, 1.52)	0.45 (0.33, 0.62)	0.41 (0.36, 0.46)	0.04 (-0.08, 0.21)
Functional dyspepsia	0.82 (0.61, 1.10)	0.47 (0.35, 0.63)	0.57 (0.52, 0.63)	-0.10 (-0.22, 0.06)
Acute gastritis	0.86 (0.57, 1.28)	0.27 (0.18, 0.40)	0.32 (0.28, 0.36)	-0.05 (-0.13, 0.09)
Irritable bowel syndrome	1.02 (0.59, 1.62)	0.44 (0.32, 0.61)	0.29 (0.25, 0.33)	0.05 (-0.07, 0.22)
Cholangitis	0.79 (0.38, 1.63)	0.08 (0.04, 0.16)	0.10 (0.07, 0.12)	-0.02 (-0.06, 0.06)
**Signs and symptoms**	0.98 (0.92, 1.04)	14.86 (14.02, 15.76)	15.22 (14.90, 15.54)	-0.36 (-1.20, 0.54)
Constipation	0.98 (0.90, 1.07)	6.25 (5.74, 6.81)	6.39 (6.20, 6.59)	-0.14 (-0.65, 0.42)
Abdominal pain	1.00 (0.92, 1.09)	7.23 (6.68, 7.84)	7.21 (7.00, 7.41)	0.03 (-0.53, 0.63)
Diarrhea	0.95 (0.86, 1.05)	4.66 (4.23, 5.14)	4.90 (4.73, 5.07)	-0.24 (-0.68, 0.24)
Vomiting	1.07 (0.83, 1.39)	0.69 (0.54, 0.89)	0.65 (0.59, 0.71)	0.05 (-0.11, 0.25)
Bloating	1.07 (0.80, 1.43)	0.57 (0.43, 0.75)	0.53 (0.48, 0.59)	0.04 (-0.10, 0.22)
**Coagulation studies**	1.00 (0.97, 1.03)	53.64 (52.16, 55.16)	53.63 (53.09, 54.18)	0.00 (-1.48, 1.52)
PT>13s	1.00 (0.97, 1.04)	44.56 (43.22, 45.95)	44.45 (43.95, 44.95)	0.11 (-1.23, 1.50)
INR>1	1.01 (0.97, 1.06)	25.00 (24.00, 26.04)	24.70 (24.33, 25.07)	0.30 (-0.70, 1.34)
PTT>35s	1.02 (0.98, 1.07)	22.99 (22.02, 24.01)	22.53 (22.17, 22.88)	0.47 (-0.50, 1.48)
**Liver and biliary tree function tests**	0.98 (0.97, 1.00)	204.47 (201.35, 207.64)	207.58 (206.42, 208.75)	-3.11 (-6.24, 0.05)
Albumin<3.5 g/dL	0.98 (0.95, 1.00)	61.55 (59.96, 63.18)	62.98 (62.38, 63.58)	-1.43 (-3.02, 0.20)
ALT>35 U/L	0.98 (0.96, 1.01)	75.44 (73.62, 77.30)	76.76 (76.08, 77.44)	-1.32 (-3.14, 0.54)
Total protein<6.0 g/dL	0.98 (0.95, 1.01)	46.53 (45.19, 47.91)	47.36 (46.86, 47.86)	-0.83 (-2.17, 0.56)
AST>35 U/L	0.99 (0.96, 1.02)	50.90 (49.45, 52.39)	51.55 (51.01, 52.09)	-0.65 (-2.10, 0.84)
LDH>100 U/L	0.98 (0.94, 1.02)	27.48 (26.42, 28.58)	28.05 (27.65, 28.44)	-0.57 (-1.63, 0.54)
CRP>0.8 mg/dL	0.97 (0.93, 1.02)	25.34 (24.33, 26.38)	26.02 (25.64, 26.40)	-0.68 (-1.69, 0.36)
ALP>92 U/L	0.99 (0.96, 1.03)	43.49 (42.17, 44.85)	43.81 (43.32, 44.30)	-0.32 (-1.64, 1.04)

Outcome*	Hazard Ratio (95% CI)†	Influenza vaccination on even days burden per 1000 persons at 12 months (95% CI) †	Influenza vaccination on odd days burden per 1000 persons at 12 months (95% CI) †	Absolute burden difference per 1000 persons at 12 months (95% CI) †
	Influenza vaccination on even days vs odd days			
Total bilirubin>1.2 mg/dL	0.99 (0.96, 1.03)	42.36 (41.05, 43.71)	42.62 (42.13, 43.11)	-0.26 (-1.57, 1.10)
GGT>30 U/L	0.99 (0.93, 1.04)	14.41 (13.66, 15.21)	14.58 (14.30, 14.87)	-0.17 (-0.92, 0.62)
Direct bilirubin>0.3 mg/dL	1.00 (0.92, 1.08)	7.37 (6.84, 7.95)	7.41 (7.21, 7.61)	-0.03 (-0.56, 0.54)
Lipase>300 U/L	1.00 (0.92, 1.07)	8.02 (7.46, 8.62)	8.05 (7.84, 8.26)	-0.03 (-0.59, 0.57)
Amylase>390 U/L	1.07 (0.91, 1.25)	1.66 (1.42, 1.95)	1.56 (1.47, 1.65)	0.10 (-0.14, 0.39)
**Any gastrointestinal outcome**	0.98 (0.96, 1.01)	216.80 (212.75, 220.92)	219.85 (218.33, 221.36)	-3.04 (-7.10, 1.08)

*. Outcomes were ascertained from day 30 after the initial positive COVID-19 test result until end of follow up

†. Adjustment through inverse probability weighting using predefined and algorithmically selected high-dimensional variables.

CI, confidence interval; GERD, gastroesophageal reflux disease; PUD, peptic ulcer disease; IBS, irritable bowel syndrome; PT, prothrombin time; INR, international normalized ratio; PTT; partial thromboplastin time; ALT, alanine transaminase; AST, aspartate transaminase; LDH, lactose dehydrogenase; CRP, c-reactive peptide; ALP, alkaline phosphatase; GGT, gamma-glutamyl transferase.
